# Determinants of Household-Level Double Burden of Malnutrition in South and Southeast Asia: A Systematic Review and Meta-analysis

**DOI:** 10.1016/j.cdnut.2026.107683

**Published:** 2026-04-01

**Authors:** Ashis Talukder, Matthew Kelly, Md Abu Sayeed, Darren Gray, Haribondhu Sarma

**Affiliations:** 1 National Centre for Epidemiology and Population Health, College of Health and Medicine, The Australian National University, Canberra, ACT, Australia; 2 Statistics Discipline, Science Engineering and Technology School, Khulna University, Khulna-9208, Bangladesh; 3 Population Health Department, QIMR Berghofer Medical Research Institute, Brisbane, QLD, Australia

**Keywords:** double burden of malnutrition, household, systematic review, meta-analysis, South Asia, Southeast Asia

## Abstract

**Background:**

The double burden of malnutrition (DBM), defined as the coexistence of undernutrition and overnutrition within the same household, is an increasing public health concern in South and Southeast Asia, yet evidence on its household-level determinants remains fragmented.

**Objectives:**

This systematic review and meta-analysis aimed to synthesize evidence on factors associated with household-level DBM in South and Southeast Asia.

**Methods:**

PubMed, Scopus, and Web of Science were searched for observational studies published between January 2000 and September 2025. Two reviewers (AT & MAS) independently screened studies, extracted data, and assessed quality using the Newcastle–Ottawa Scale adapted for cross-sectional studies. Random-effects meta-analyses were conducted for factors reported in ≥5 studies, and pooled odds ratios (ORs) with 95% confidence intervals (CIs) were estimated.

**Results:**

Thirty studies were included, of which 26 were eligible for meta-analysis. Urban residence (OR = 1.38, 95% CI: 1.20, 1.59), higher household wealth (OR = 1.55, 95% CI: 1.31, 1.83), older maternal age (OR = 2.22, 95% CI: 1.97, 2.50), maternal short stature (OR = 1.90, 95% CI: 1.69, 2.13), older child age (OR = 1.79, 95% CI: 1.44, 2.23), and cesarean delivery (OR = 1.76, 95% CI: 1.20, 2.57) were associated with higher likelihood of DBM. Higher maternal education and breastfeeding were found to be protective factors.

**Conclusions:**

Effective interventions should adopt integrated, life-course approaches that simultaneously address undernutrition and overnutrition across critical stages, particularly during adolescence, pregnancy, and early childhood. Policies should prioritize maternal education, breastfeeding promotion, and adolescent nutrition to break intergenerational cycles of malnutrition.

This study was registered at PROSPERO as CRD420251155844.

## Introduction

The double burden of malnutrition (DBM) is a growing public health concern in South and Southeast Asia, where undernutrition, such as stunting, wasting, and micronutrient deficiencies, occurs alongside rising rates of overweight, obesity, and diet-related noncommunicable diseases [1,2]. This paradoxical coexistence has emerged as countries undergo rapid demographic and socioeconomic transitions that reshape dietary patterns and lifestyles [3]. As a result, many households face both forms of malnutrition simultaneously. Limited household resources, unequal food distribution, and poor diet quality combine with reduced physical activity to create conditions where children remain undernourished, whereas adults gain excess weight [4,5].

Early-life malnutrition also carries profound consequences for child development. Stunting during the first 1000 d of life impairs early brain development, reduces cognitive performance, and is associated with lower educational attainment and diminished economic productivity in adulthood [[6], [7], [8]]. It is estimated that >200 million children aged <5 y in low- and middle-income countries are failing to reach their developmental potential as a direct consequence of undernutrition, poverty, and inadequate caregiving environments [6]. The consequences extend beyond immediate health concerns, as stunted children often become short-statured mothers who are more likely to deliver low-birth-weight infants with increased risk of growth faltering, thereby transmitting malnutrition risk to the next generation and threatening long-term development progress across the region [4].

The magnitude of this problem is particularly alarming in South and Southeast Asia, which together account for ∼54% of the global burden of child stunting, while simultaneously experiencing the fastest rates of increase in adult overweight and obesity [9]. In Bangladesh, Nepal, and India, DBM affects between 8% and 14% of households, where undernourished children and mothers with overweight or obesity coexist under the same roof [5,10]. In various Indian districts, school-aged children experience high rates of both undernutrition and overnutrition at the population level, with notable gender disparities in the distribution of these conditions [11]. These 2 regions share critical characteristics that make them ideal for comparative analysis: they are home to over one-third of the world’s population, have experienced rapid but uneven economic growth, are undergoing accelerated nutrition transition, yet continue to face persistent poverty and food insecurity [3,[12], [13], [14]]. These epidemiological trends are systematic manifestations of broader societal transformations. Globalization and rapid urbanization have fundamentally altered food environments throughout the region, increasing availability and affordability of processed foods laden with fats, sugars, and refined carbohydrates while simultaneously transforming occupational structures toward more sedentary work [3,13]. Yet amid these changes, poverty and food insecurity persist, continuing to restrict access to the diverse, nutrient-dense foods necessary for optimal child growth and development [14]. The consequence is a widening nutritional inequality where economic progress and urbanization do not automatically translate into improved health outcomes for all household members.

These epidemiological patterns are shaped by profound socioeconomic inequities in the distribution of undernutrition and overnutrition. An analysis of 80 countries shows that stunting among children aged <5 y is inversely associated with family wealth, with the poorest families bearing the greatest burden, particularly in South Asia [15]. In contrast, overweight children are more common among wealthier households in low- and middle-income countries, although obesity rates are increasing more rapidly among socioeconomically vulnerable populations [15]. This creates a double inequity at the household level, where poorer families face persistent child undernutrition alongside rising adult overweight, whereas wealthier households experience growing overnutrition even as pockets of child undernutrition persist [13]. The intergenerational transmission of stunting and obesity is embedded in these socioeconomic structures, including food insecurity, gender inequity, and limited access to healthcare and education, indicating that nutrition-specific interventions alone cannot break these cycles [15,16].

These population-level patterns manifest clearly at the household level, which acts as the fundamental unit for understanding and addressing DBM. Within households, the mother–child dyad represents an important relationship, as maternal nutritional status, knowledge, and behaviors exert profound influences on children’s growth trajectories [17,18]. However, complex intrahousehold dynamics, shaped by economic resources, structures, educational attainment, and cultural norms, determine how food and care are distributed among family members [19,20]. Among these factors, maternal education stands out as an important determinant, influencing nutrition knowledge, child feeding practices, dietary choices, and healthcare-seeking behaviors [13,21]. Mothers with higher education demonstrate greater capacity to navigate complex and changing food environments, making informed decisions that help prevent child undernutrition while avoiding their own overnutrition [13,22]. Effective interventions must therefore address these interconnected household dynamics rather than treating child and maternal malnutrition as isolated problems.

**FIGURE 1:**
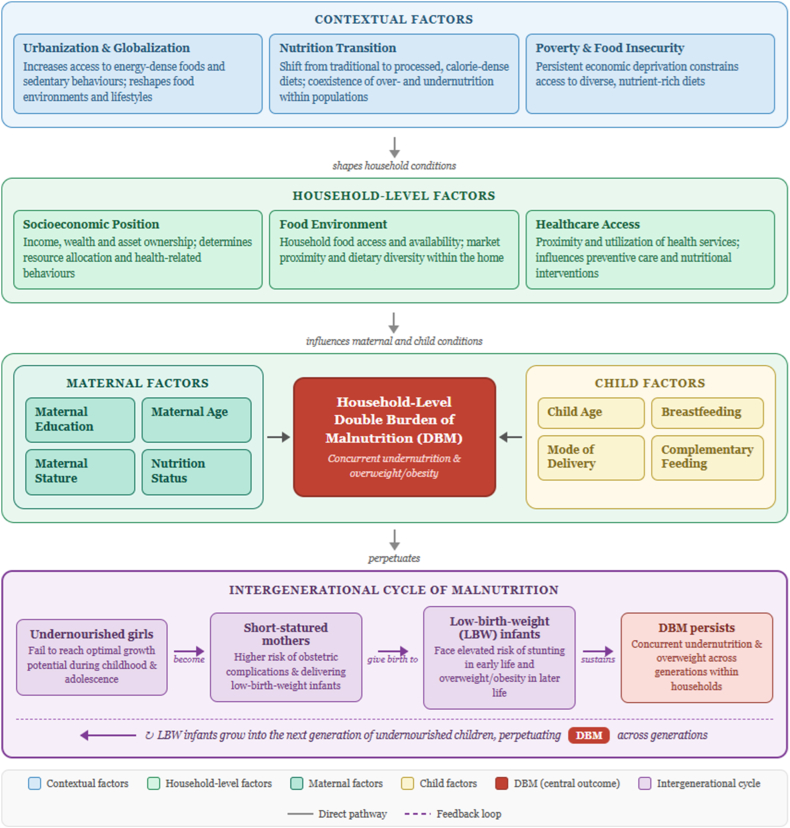
Conceptual framework illustrating the hypothesized pathways through which contextual, household-level, maternal, and child factors contribute to DBM within households in South and Southeast Asia. Solid arrows represent direct hypothesized associations; the curved feedback arrow denotes the intergenerational cycle of malnutrition. DBM, double burden of malnutrition; LBW, low-birth-weight.

Drawing on the established conceptual pathways described above, this review is guided by a conceptual framework that organizes the determinants of household-level DBM across 3 interrelated domains—contextual, household and maternal, and child-level factors—operating within an intergenerational cycle of malnutrition. At the contextual level, urbanization, economic growth, and globalization reshape food environments and lifestyles by increasing access to energy-dense foods and sedentary behaviors, whereas persistent poverty and food insecurity constrain access to diverse, nutrient-rich diets. At the household and maternal level, socioeconomic position, maternal education, age, and stature influence resource allocation and health-related behaviors, with maternal education playing a central role in shaping nutrition knowledge, feeding practices, dietary choices, and healthcare utilization. At the child level, factors such as age, breastfeeding practices, and mode of delivery directly affect growth trajectories and nutritional outcomes, with suboptimal feeding practices increasing the likelihood of undernutrition. These domains are interconnected through an intergenerational pathway, whereby undernourished girls are more likely to become short-statured mothers and give birth to low-birth-weight infants, thereby perpetuating DBM across generations (Figure 1).

Despite growing recognition of DBM as a critical public health priority in South and Southeast Asia, significant gaps persist in the understanding of its household-level determinants. Although numerous studies have investigated potential risk factors across sociodemographic, economic, dietary, behavioral, and environmental domains, the evidence base remains fragmented and inconsistent [10,[23], [24], [25], [26]]. These inconsistencies stem from 2 main sources. First, genuine contextual heterogeneity exists across countries at different stages of nutritional transition, whereby the same determinants, such as household wealth or urbanization show divergent associations depending on the local economic and food environment, as evidenced by differing findings across Myanmar [23], India [25], and Bangladesh, Nepal, Pakistan, and Myanmar collectively [26]. Second, substantial methodological variation limits comparability, including differences in the operational definition of DBM. Some studies restrict the definition to mothers with overweight or obesity with stunted children [23,24] whereas others apply broader criteria encompassing additional malnutrition combinations [25,26]. Variation in sample selection and covariate adjustment strategies further compounds this problem. Together, these contextual and methodological sources of variation produce a scattered evidence base that is difficult to interpret and challenging to translate into coherent policy guidance.

An important contribution to this field was made by Biswas et al. [24], who drew on large, harmonized Demographic and Health Survey (DHS) data from multiple countries in South and Southeast Asia to characterize the patterns and determinants of household-level DBM across the region. That study provided valuable cross-national evidence on the sociodemographic correlations of DBM and established a foundational understanding of how household and maternal characteristics relate to the coexistence of child undernutrition and maternal overweight. However, by relying on a single harmonized dataset and a predominantly descriptive analytical framework, it was not designed to systematically pool effect estimates across independently conducted studies or to formally quantify the magnitude and consistency of associations identified in the broader primary literature. Consequently, several important questions remain unresolved. First, when the full body of independently conducted studies is considered, how robust and generalizable are the associations identified using a single harmonized survey dataset? Second, do the direction and magnitude of key associations vary systematically with the geographic subregion examined or the operational definition of DBM used? Third, given the rapidly evolving nutritional landscape across South and Southeast Asia, do more recently published studies corroborate or challenge the conclusions of that earlier work?

To address these critical knowledge gaps, we conducted a comprehensive systematic review and meta-analysis to synthesize evidence on household-level factors associated with DBM in South and Southeast Asia. Although a previous systematic review examined the global prevalence and predictors of household-level DBM [27], its focus was mainly on methodological heterogeneity and prevalence patterns and did not conduct a meta-analysis or specifically synthesize household-level determinants in South and Southeast Asia. In addition, a recent systematic review and meta-analysis assessed DBM among mother–child pairs in Ethiopia [28], but its findings were restricted to a single-country context. No previous review has comprehensively synthesized household-level factors associated with DBM specifically across the diverse contexts of South and Southeast Asia, where the burden is particularly high and rapidly evolving [5]. Through systematic identification of relevant studies, rigorous quality assessment, and pooling effect estimates using meta-analytical techniques, we sought to identify consistent patterns across diverse settings and populations. This review was designed to answer 2 fundamental questions: first, what household-level factors show consistent associations with DBM across South and Southeast Asian contexts? Second, how strong are these associations, and do they vary systematically by geographical region or operational definition of DBM? By providing a comprehensive synthesis of household-level determinants of DBM in this region, this review aimed to establish a robust evidence foundation for designing integrated nutrition policies and programs capable of breaking the cycle of dual malnutrition that threatens the health and prosperity of millions of families across South and Southeast Asia.

## Methods

We conducted this systematic review following the PRISMA guidelines to ensure a rigorous and transparent approach. The review was prospectively registered with PROSPERO (CRD420251155844), and the completed PRISMA checklist outlining all stages of the review process is provided in Supplementary File.

### Study selection and eligibility criteria

Observational and longitudinal studies that investigated factors associated with DBM at the household level were included according to our research questions and guided by population, intervention/exposure, comparator, outcomes, and study design framework (Table 1).

**TABLE 1:** PICOS criteria for inclusion of studies.

Component	Inclusion criteria
P	Households or mother–child pairs in South Asia (Afghanistan, Bangladesh, Bhutan, India, Iran, Maldives, Nepal, Pakistan, and Sri Lanka) and Southeast Asia (Brunei, Cambodia, Indonesia, Laos, Malaysia, Myanmar, Philippines, Singapore, Thailand, Timor-Leste, and Vietnam).
I	Sociodemographic, economic, dietary, behavioral, environmental, and maternal or child characteristics that may be associated with DBM (e.g., household wealth, parental education, dietary diversity, urban or rural residence).
C	Not applicable.
O	DBM defined at household level (e.g., mother overweight/obese and child stunted/wasted/underweight; household with undernourished and overnourished members).
S	Quantitative observational studies (cross-sectional, case-control, cohort) and longitudinal studies that investigated factors associated with DBM at the household level.

Abbreviations: DBM, double burden of malnutrition; PICOS, Population, Intervention/Exposure, Comparator, Outcomes, Study Design.

### Exclusion criteria

Studies were excluded if they were conducted outside South and Southeast Asia or focused on only a single form of malnutrition, such as stunting, wasting, underweight, or overweight/obesity. We also excluded studies conducted solely at the population or national level, without disaggregated household- or mother–child-level data. Additionally, reviews, editorials, commentaries, conference abstracts without full data, study protocols, and letters were not considered. Studies lacking sufficient information on risk factors of DBM, as well as publications in languages other than English, were also excluded.

### Search strategy

We conducted a comprehensive literature search in 3 major electronic databases: PubMed, Scopus, and Web of Science for studies published between 1 January, 2000 and 15 September, 2025. The year 2000 was chosen as the starting point because the concept of DBM began gaining prominence in global health research around this time [29]. To identify additional relevant studies not captured in the database search, grey literature and reference lists of included articles were also hand-searched. The search strategy incorporated key terms, including “double burden of malnutrition,” “households,” “mother–child pair,” and “risk factors.” Detailed search strategies for each database are provided in Supplementary Table 1.

### Study screening and data extraction

Study screening and data extraction were conducted independently and in duplicate to minimize the risk of error and bias. Two reviewers (AT and MAS) independently screened all retrieved records at the title and abstract stage to assess relevance, followed by an independent full-text review of potentially eligible studies to confirm eligibility. Any disagreements at either screening stage were resolved through discussion or, when necessary, by consultation with a third reviewer (HS). Data extraction was subsequently performed independently by the same 2 reviewers (AT and MAS). Each reviewer extracted data from all included studies separately, without access to the other reviewer’s extractions. The extracted information included first author, year of publication, country or region, study design, sample size, study population, definitions used to identify household-level DBM, outcome measures, exposure factors examined, and the main findings on associations between these factors and DBM. Once both reviewers had completed independent extractions for all included studies, the 2 sets of extracted data were compared systematically, and any discrepancies were identified and resolved through discussion and cross-checking against the original study reports. Where disagreement persisted, the third reviewer (HS) provided a final decision to ensure consistency and accuracy across all extracted data.

### Risk of bias assessment

Two independent reviewers assessed the risk of bias for each included study using a modified version of the Newcastle–Ottawa Scale adapted for cross-sectional studies (NOS-xs) [30]. The adapted scale comprised 3 domains: *1*) study sample selection (maximum 2 stars), *2*) assessment of exposure and outcome (maximum 4 stars), and *3*) confounding factors (maximum 3 stars), with a total possible score of 9 stars. The assessment evaluated the representativeness of the study sample, the adequacy of the sample size, the validity and reliability of the measurement tools for exposure and outcome, and the appropriate control and measurement of confounders. Each item within the domains was rated according to predefined criteria, and a single best answer was selected per item. Studies scoring 7–9 stars were considered to have a low risk of bias (good quality), 5–6 stars indicated a moderate risk of bias (satisfactory quality), and 0–4 stars reflected a high risk of bias (unsatisfactory quality) [30]. Any discrepancies between reviewers were resolved through discussion and consensus.

### Statistical methods

We used Microsoft Excel 2019 to organize our data and RStudio (Posit Software, PBC; R version 4.3.1) to perform the analyses. To address our first research question on identifying factors associated with DBM, we calculated odds ratios (ORs) with 95% confidence intervals (CIs) as the summary measures of effect. Although not all factors were reported in every study, meta-analyses were conducted for those factors reported in ≥5 studies with extractable raw data. For studies that examined multiple forms of child undernutrition (stunting, wasting, or underweight) in combination with maternal overweight/obesity, we extracted data for each DBM definition separately. When studies reported results for more than 1 DBM definition, we included all available combinations in the overall meta-analysis and subsequently conducted subgroup analyses based on DBM definition categories: *1*) overweight mother with stunted child (OWM + SC), and *2*) composite definitions that included overweight mother with stunted, wasted, or underweight child (OWM + SC/WS/UW).

To address our second research question regarding how associations vary by strength and geographic location, we performed subgroup analyses stratified by: *1*) DBM definition (OWM + SC compared with composite definitions), and *2*) geographic region (South Asia compared with Southeast Asia). For each subgroup, we calculated pooled OR and 95% CIs separately and compared effect sizes across subgroups. Factors not suitable for meta-analysis due to insufficient data (<5 studies) were summarized qualitatively in a narrative synthesis.

Heterogeneity was assessed for clinical, methodological, and statistical differences using the *I*^2^ statistic and Q-test (*P* value). Given the expected heterogeneity across diverse populations and settings, a random-effects model was applied for all meta-analyses using the DerSimonian–Laird method. Interpretation of *I*^2^ values followed standard guidelines: 0%–40% indicating low heterogeneity, 30%–60% moderate heterogeneity, 50%–90% substantial heterogeneity, and 75%–100% considerable heterogeneity.

To evaluate the robustness of our findings, we performed leave-1-out sensitivity analyses by sequentially removing each study and recalculating the pooled effect estimates. This approach assessed whether any single study disproportionately influenced the overall results. Publication bias was assessed using Egger’s regression test and visual inspection of funnel plots for asymmetry. A *P* value < 0.05 in Egger’s test was considered indicative of potential publication bias.

All statistical analyses were conducted using the “meta” and “metafor” packages in R. Forest plots were generated to visualize pooled effect estimates and their CIs for each factor, and funnel plots were created to assess publication bias.

### Ethical consideration

No ethical approval is required as we are doing this research based on published papers.

## Results

### Overview of the literature screening process

**FIGURE 2:**
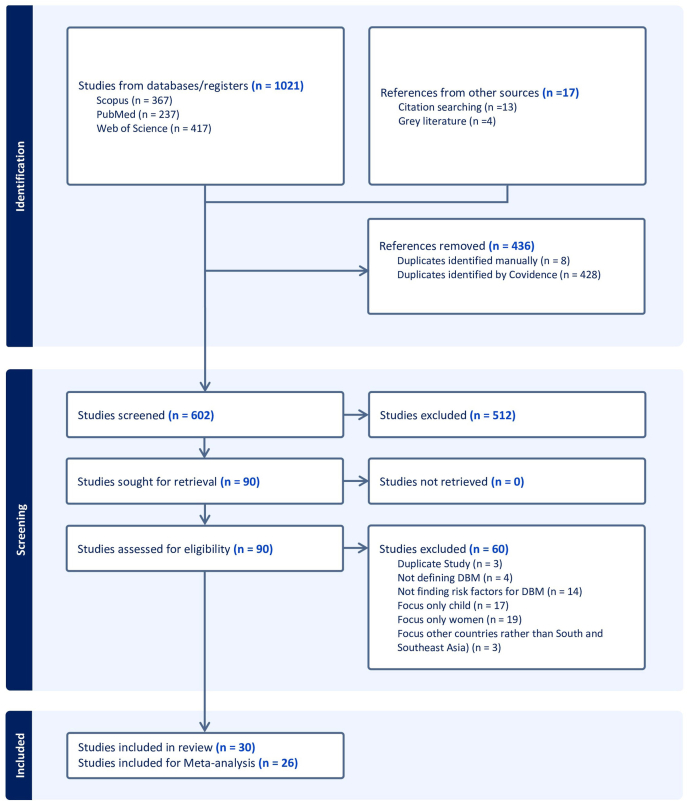
PRISMA flow diagram illustrating the selection process of studies included in the systematic review.

A total of 1021 records were identified through 3 database searches (PubMed = 237, Scopus = 367, and Web of Science = 417) and other sources such as citation searching and grey literature (*n* = 17). After removing 436 duplicate records (8 manually and 428 using Covidence software), 602 studies remained for title and abstract screening. Of these, 512 studies were excluded as they did not meet the inclusion criteria (Figure 2). The full texts of 90 studies were reviewed for eligibility, and 60 were excluded for reasons such as focusing only on women or children, failing to define DBM, failing to assess associated factors, or being conducted outside South and Southeast Asia. Finally, 30 studies [10,13,[23], [24], [25], [26],[31], [32], [33], [34], [35], [36], [37], [38], [39], [40], [41], [42], [43], [44], [45], [46], [47], [48], [49], [50], [51], [52], [53], [54]] met the inclusion criteria and were included in this systematic review, of which 26 were eligible for meta-analysis (the list of articles is available in Table 2) as we were able to extract raw data from these studies. We were unable to extract raw data from 4 studies because the data were presented in an unsuitable format; therefore, these studies were excluded from the meta-analysis. The selection process is summarized in Figure 2, which presents the PRISMA flow diagram of study inclusion.

**TABLE 2:** Characteristics of included studies and distribution of selected 12 sociodemographic factors across study populations.

Study (author, year)	Country	Data collection period (related survey)	Sample size (*n*)	Effectmeasure	Household-level factors (2)			Parental-level factors (5)					Child-level factors (2)		Behavioral and healthcare factors (3)		
					Place of residence urban vs. rural	Wealth status rich vs. poor		Maternal age ≥25 y vs. 15–24 y	Maternal stature <150 cm vs. ≥150 cm	Maternal education high (s/higher) vs. low (none/primary)	Paternal education high (s/higher) vs. low (none/primary)	Maternal working status yes (employed) vs. no	Child age 24–59 mo vs. 0–23 mo	Child sex male vs. female	Breastfeeding status yes vs. no	C-section yes vs. no (vaginal)	Media exposure yes vs. no
					Urban (%)	Rich (%)	Poor (%)	≥25 y (%)	<150 cm (%)	High (%)	High (%)	Yes (%)	24–59 mo (%)	Male (%)	Yes (%)	Yes (%)	Yes (%)
**Anik et al. (2019)** [26]	**Bangladesh**	**2014 (BDHS)**	**6478**	**Adjusted OR**	**25.8**	**40.2**	**39.1**	**51.5**	—	**57.1**	—	**25.5**	**54.2**	**52.7**	**56.6**	—	**37.2**
Anik et al. (2019)	**Nepal**	**2016 (NDHS)**	**2670**	**Adjusted OR**	**53.9**	**35.1**	**42.9**	**51.6**	—	**50.3**	—	**46.1**	**28.1**	**53.4**	**92.7**	—	**77.5**
Anik et al. (2019)	**Pakistan**	**2012–13 (PDHS)**	**5770**	**Adjusted OR**	**30.2**	**35.2**	**45.9**	**79.9**	—	**27.1**	—	**26.2**	**45.1**	**52.5**	**50.7**	—	**45.4**
Anik et al. (2019)	**Myanmar**	**2015–16 (MDHS)**	**3541**	**Adjusted OR**	**22.9**	**33.9**	**48.8**	**81.7**	—	**38.5**	—	**56.8**	**52.9**	**51.2**	**54.9**	—	**82.7**
**Das et al. (2018)** [32]	**Bangladesh**	**2014 (BDHS)**	**5951**	**Adjusted OR**	—	**41.0**	**39.2**	**49.6**	—	**58.1**	**46.4**		—	—	—	**24.0**	**36.5**
**Gaupholm et al. (2023)** [34]	**Philippines**	**2013 (NNS)**	**5837**	**Adjusted OR**	—	**37.1**	**42.1**	—	—	**15.7**	—	**42.48**	—	—	—	—	—
**Hauqe et al. (2017)** [35]	**Bangladesh**	**2014 (BDHS)**	**5697**	**Adjusted OR**	**26.0**	**40.3**	**39.4**	**52.1**	—	—	—	**26.1**	**52.7**	—	—	—	—
**Hong et al. (2020)** [23]	**Myanmar**	**2015–16 (MDHS)**	**5687**	**Adjusted OR**	**21.8**	**35.3**	**32.3**	**81.9**	**31.4**	**36.2**	—			—	—	—	—
**Ihab et al. (2013)** [36]	**Malaysia**	**2012 (primary data)**	**223**	**Unadjusted OR**	—	—	—	—	—	**66.0**	—	**69.0**	—	—	—	—	—
**Jayalakshmi et al. (2019)** [37]	**India**	**2012 IHDS-II**	**344**	**Unadjusted OR**	**56.4**	—	—	—	—	—	**33.7**	—	—	—	—	—	—
**Khaliq et al. (2025)** [38]	**Pakistan**	**2017–18 (PDHS)**	**6198**	**Adjusted OR**	**45.1**	**35.4**	**45.2**	—	—	**34.2**	—	**15.6**	—	**48.7**	—	**17.5**	—
**Krismanita et al. (2022)** [40]	**Indonesia**	**2019 (primary data)**	**274**	**Adjusted OR & Unadjusted OR**	—	**34.7**	**65.3**	—	**47.1**	**21.9**	**42.3**	—	—	—	**35.8**	—	—
**Limon et al. (2021)** [41]	**Bangladesh**	**2017–18 (BDHS)**	**3772**	**Adjusted OR**	**33.3**	**37.2**	**41.5**	—	—	**62.3**	**42.9**	—	—	—	—	—	—
**Mahmudiono et al. (2018)** [54]	**Indonesia**	**2013 (RISKESDAS)**	**685**	**Adjusted OR**	—	—	—	—	—	**53.3**	—	—	—	—	—	—	—
**Nakphong et al. (2021)** [42]	**Cambodia**	**2014 (CDHS)**	**14,988**	**Adjusted OR**	—	**22.2**	**33.4**	**75.7**	**30.6**	—	—	**66.4**	**60.1**	—	—	—	—
**Oddo et al. (2012)** [43]	**Indonesia**	**2007 (IDHS)**	**247,126**	**Adjusted OR**	—	**32.6**	**43.7**	**68.4**	**51.6**	**36.1**	—	—	—	**51.0**	**55.2**	—	—
Oddo et al. (2012)	**Bangladesh**	**2004 (BDHS)**	**168,317**	**Adjusted OR**	—	**29.3**	**45.2**	**60.7**	**45.4**	**29.9**	—	—	—	**52.0**	**65.3**	—	—
**Patel et al. (2020)** [25]	**India**	**2015–16 (NFHS-4)**	**184,680**	**Adjusted OR**	**29.1**	**34.9**	**45.1**	**66.0**	—	**58.2**	—	—	**62.0**	**52.9**	**46.7**	**18.4**	—
**Rachmah et al. (2021)** [44]	**Indonesia**	**2018 (primary data)**	**436**	**Adjusted OR**	—	—	—	—	—	**61.9**	**69.4**	**25.7**	—	—	—	—	—
**Rahman et al. (2021)** [45]	**Bangladesh**	**2017–18 (BDHS)**	**7662**	**Adjusted OR**	**30.9**	**38.9**	**41.7**	**85.2**	—	**56.0**	**44.0**	—	—	—	**58.2**	**15.3**	**49.6**
**Ramasubramani et al. (2024)** [10]	**India**	**2019–21 (NFHS-5)**	**167,380**	**Adjusted OR**	**26.5**	**30.5**	**49.8**	**89.2**	—	**67.2**	—	—	**67.8**	**51.7**	**61.9**	**21.5**	—
**Saibul et al. (2009)** [46]	**Malaysia**	**2002–05 (Primary Data)**	**227**	**Unadjusted OR**	—	—	—	—	—	—	—	**24.7**	—	—	—	—	—
**Sarker et al. (2022)** [47]	**Bangladesh**	**2017–18 (BDHS)**	**8697**	**Adjusted OR**	—	**38.3**	**44.8**	**75.2**	—	—	**49.4**	**40.8**	—	—	—	—	**42.4**
**Sekiyama et al. (2015)** [48]	**Indonesia**	**2006-07 (ENVRERA Project)**	**242**	**Unadjusted OR**	—	—	—	—	**54.2**	**21.5**	—	**24.7**	—	—	—	—	—
**Sengupta et al. (2025)** [49]	**India**	**2019–21 (NFHS-5)**	**360**	**Adjusted OR**	—	—	—	—	—	**56.1**	**35.0**	—	—	**51.1**	—	—	—
**Shariff et al. (2024)** [50]	**Malaysia**	**2017– 19 (Primary Data)**	**451**	**Adjusted OR**	—	—	—	—	—	**5.7**	**39.5**	**16.2**	**54.8**	**49.9**	—	—	—
**Singh et al. (2023)** [52]	**India**	**2019–21 (NFHS-5)**	**122,922**	**Adjusted OR**	**26.4**	**35.2**	**45.2**	**60.3**	—	**69.3**	—	—	—	**52.2**	**81.2**	**23.6**	—
**Sunuwar et al. (2020)** [51]	**Nepal**	**2016 (NDHS)**	**2261**	**Adjusted OR**	—	**35.9**	**42.1**	**58.9**	**10.0**	**46.8**	**62.1**	**59.6**	**58.3**	**52.7**	**78.8**	**9.8**	—
**Sutopa et al. (2022)** [53]	**Bangladesh**	**2017–18 (BDHS)**	**4377**	**Adjusted OR**	**33.5**	**37.0**	**42.5**	**45.5**	—	**57.9**	**22.4**	**22.6**	—	—	**88.0**	**23.0**	**64.6**
**Talukder et al. (2024)** [13]	**Bangladesh**	**2017–18 (BDHS)**	**7718**	**Adjusted OR**	**35.0**	**39.2**	**42.2**	—	—	**64.1**	—	—	—	**51.9**	**60.2**	—	—
Talukder et al. (2024)	**Cambodia**	**2014 (CDHS)**	**3836**	**Adjusted OR**	**35.3**	**34.0**	**45.3**	—	—	**74.7**	—	—	—	**51.1**	**36.3**	—	—
Talukder et al. (2024)	**Nepal**	**2016 (NDHS)**	**2596**	**Adjusted OR**	**49.4**	**36.6**	**42.9**	—	—	**71.0**	—	—	—	**50.2**	**65.8**	—	—
Talukder et al. (2024)	**Timor-Leste**	**2016 (TDHS)**	**6309**	**Adjusted OR**	**29.2**	**38.7**	**45.8**	—	—	**55.8**	—	—	—	**51.7**	**53.4**	—	—

Data presentation: Each cell reports the percentage (%) distribution of the several categories of the selected covariates within the total analytic sample for that study. Values represent sample proportions, not effect sizes.

Effect measure: adjusted OR, adjusted odds ratio; unadjusted OR, crude OR.

— (em dash): Indicates that the proportion of the factors was not able to extract or not reported in that study.

Variable categories (consistent with Table 3):

• Place of residence: Urban (%) compared with rural.

• Wealth status (Wealth Index): Studies used wealth index factor scores derived from household asset ownership. The wealth index was operationalized in 2 ways across the included literature:

Five-category scale (poorest/poorer/middle/richer/richest): To ensure comparability, the 2 lowest quintiles (poorest + poorer) were merged to form the “Poor” group and the 2 highest quintiles (richer + richest) were merged to form the “Rich” group. The middle quintile was excluded from the binary comparison.

Three-category scale (poor/middle/rich): The “Poor” and “Rich” categories were used directly as reported, with the middle category excluded from the binary comparison.

• Maternal age: ≥25 y vs. 15–24 y

• Maternal stature: <150 cm vs. ≥150 cm

• Maternal education: high = secondary or higher vs. low = none/primary

• Paternal education: high = secondary or higher vs. low = none/primary

• Maternal working status: yes = employed vs. no = not employed

• Child age: 24–59 mo vs. 0–23 mo

• Child sex: male vs. female

• Breastfeeding status: yes = currently breastfeeding vs. no

• C-section delivery: yes vs. no = vaginal delivery

• Media exposure: yes = exposed to TV/radio/newspaper vs. no

Abbreviations: BDHS, Bangladesh Demographic and Health Survey; CDHS, Cambodia Demographic and Health Survey; ENVRERA, Environmental Research in Rural Asia; IDHS, Indonesia Demographic and Health Survey; IHDS-II, India Human Development Survey, Wave 2; MDHS, Myanmar Demographic and Health Survey; NDHS, Nepal Demographic and Health Survey; NFHS, National Family Health Survey (India); NNS, National Nutrition Survey (Philippines); PDHS, Pakistan Demographic and Health Survey; RISKESDAS, Riset Kesehatan Dasar (Indonesia National Basic Health Research); TDHS, Timor-Leste Demographic and Health Survey.

Studies included (*n* = 33 study-country observations from 26 unique publications):

Anik et al. (2019) (Bangladesh, Nepal, Pakistan, Myanmar); Das et al. (2018); Gaupholm et al. (2023); Hauqe et al. (2017); Hong et al. (2020); Ihab et al. (2013); Jayalakshmi et al. (2019); Khaliq et al. (2025); Krismanita et al. (2022); Limon et al. (2021); Mahmudiono et al. (2018); Nakphong et al. (2021); Oddo et al. (2012) [Indonesia, Bangladesh]; Patel et al. (2020); Rachmah et al. (2021); Rahman et al. (2021); Ramasubramani et al. (2024); Saibul et al. (2009); Sarker et al. (2022); Sekiyama et al. (2015); Sengupta et al. (2025); Shariff et al. (2024); Singh et al. (2023); Sunuwar et al. (2020); Sutopa et al. (2022); Talukder et al. (2024) (Bangladesh, Cambodia, Nepal, Timor-Leste).

### Characteristics of included studies

The key characteristics of studies included in the systematic review on factors associated with DBM at the household level in South and Southeast Asia are presented in Supplementary Table 2. All studies employed a cross-sectional design and covered a wide range of countries, including Bangladesh, India, Indonesia, Malaysia, Nepal, Pakistan, Philippines, Myanmar, Cambodia, and Timor-Leste. Sample sizes varied considerably, ranging from fewer than 300 households in small-scale studies to >700,000 households in large multicountry analyses. Most studies (17 studies) defined DBM as the coexistence of a mother with overweight or obesity and a stunted child (OWM + SC). However, several studies (14 studies) used broader definitions that also considered other forms of child undernutrition, such as wasting or underweight, resulting in alternative combinations like OWM+WC (overweight mother–wasted child), OWM + UWC (overweight mother–underweight child), or OWM + SC/WC/UW (overweight mother with any stunted, wasted, or underweight child). Most studies reported adjusted odds ratios as the main effect measure, whereas a few presented crude OR, or descriptive statistics. The methodological quality of the studies assessed using the NOS-xs, ranged from 7 to 9, indicating that the included studies were of good quality [30]. The NOS-xs ratings by each domain are available in Supplementary Table 3.

### Meta-analysis results of factors associated with DBM

We conducted a meta-analysis reported in ≥5 studies, for which raw data could be extracted. This analysis summarized the pooled effects of these 12 factors associated with DBM in South and Southeast Asia. These factors were grouped into 4 major categories: household-level factors, parental-level factors, child-level factors, and behavioral and healthcare-related factors. Most of the included factors exhibited moderate to high heterogeneity, reflecting contextual variations across countries in the region. Table 3 presents the definitions and categorizations of the 12 factors included in the meta-analysis. The data collection periods of the included studies, along with the distribution of these sociodemographic factors across study populations, are provided in Table 2.

**TABLE 3:** Definitions of the 12 key factors included in meta-analysis.

Category	Factor	Definition/measurement
Household level	Place of residence	Urban vs. rural household location
	Wealth status	Household wealth quintile (rich vs. poor). Studies used wealth index factor scores derived from household asset ownership. The wealth index was operationalized in 2 ways across the included literature: • Five-category scale (poorest/poorer/middle/richer/richest): to ensure comparability, the 2 lowest quintiles (poorest + poorer) were merged to form the “Poor” group and the 2 highest quintiles (richer + richest) were merged to form the “Rich” group. The middle quintile was excluded from the binary comparison. • Three-category scale (poor/middle/rich): the “Poor” and “Rich” categories were used directly as reported, with the middle category excluded from the binary comparison.
Parental level	Maternal age	Mother’s age at time of survey (≥25 vs. 15–24 y)
	Maternal stature	Mother’s height (<150 vs. ≥150 cm)
	Maternal education	Maternal educational attainment was categorized as “High” for secondary or higher education and “Low” for no or primary education.
	Paternal education	Father’s educational attainment was categorized as “High” for secondary or higher education and “Low” for no or primary education.
	Maternal working status	Whether mother was employed outside home (Yes vs. No)
Child level	Child age	Age of child in household (24–59 vs. 0–23 mo)
	Child sex	Sex of child (male vs. female)
Behavioral and healthcare	Breastfeeding status	Current breastfeeding status was assessed at the time of the survey, with children coded as “Yes” if they were currently breastfeeding and “No” otherwise.
	C-section	Among mother–child pairs where the baby was born via C-section is coded as “Yes” Otherwise “No.”
	Media exposure	Household media exposure was coded as “Yes” if the respondent watched TV, listened to the radio, or read newspapers at least once a week, and “No” otherwise.

Abbreviation: C-section, cesarean section

### Household-level factors

Urban residence and higher household wealth were both significantly associated with increased likelihood of DBM (detailed in Supplementary Figures 1–12, Figure 3). Households in urban areas were 38% more likely to experience DBM than their rural counterparts (pooled OR = 1.38, 95% CI: 1.20, 1.59; *I*^2^ = 84.4%). Similarly, households in the richest wealth quintile were 55% more likely to experience DBM compared with the poorest households (pooled OR = 1.55, 95% CI: 1.31, 1.83; *I*^2^ = 71.9%).

**FIGURE 3:**
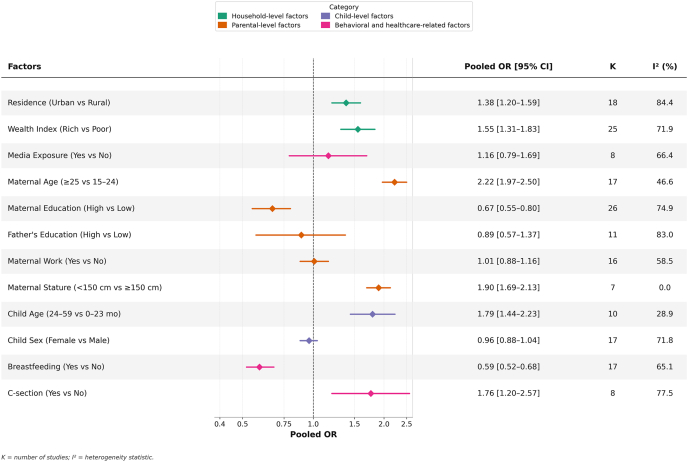
Pooled estimates of factors associated with household-level DBM in South and Southeast Asia. Last category of each factor is considered as reference category. K, number of studies to estimate the pooled estimate. Factor-specific Forest plots are available in Supplemental Figures 1–12. CI, confidence interval; DBM, double burden of malnutrition; OR, odds ratio.

### Parental-level factors

Maternal characteristics showed stronger and more consistent associations with DBM than paternal factors. Households with older mothers (≥25 y) were more than twice as likely to experience DBM compared with those with younger mothers (pooled OR = 2.22, 95% CI: 1.97, 2.50; *I*^2^ = 46.6%). Maternal short stature was also associated with 90% higher likelihood of DBM (pooled OR = 1.90, 95% CI: 1.69, 2.13; *I*^2^ = 0%). Moreover, higher maternal education reduced the odds of DBM by 33% (pooled OR = 0.67, 95% CI: 0.55, 0.80; *I*^2^ = 74.9%). On the other hand, neither paternal education nor maternal working status showed statistically significant associations with DBM.

### Child-level factors

At the child level, both age and sex were examined as predictors of DBM. Children aged 24–59 mo were 79% more likely to be in DBM-affected households compared with younger children aged 0–23 mo (pooled OR = 1.79, 95% CI: 1.44, 2.23; *I*^2^ = 28.9%). However, child sex was not significantly associated with DBM (pooled OR = 0.96, 95% CI: 0.88, 1.04; *I*^2^ = 71.8%).

### Behavioral and healthcare-related factors

Behavioral and health service–related factors also showed notable associations. Current breastfeeding status of the child emerged as a strong protective factor against DBM (pooled OR = 0.59, 95% CI: 0.52, 0.68; *I*^2^ = 65.1%), with mother–child pairs where the child was currently being breastfed at the time of survey showing lower odds of DBM compared with pairs where the child was not currently breastfed. We acknowledge that this finding is most applicable to children aged <24 mo. Also, cesarean delivery for the birth of a child was significantly associated with higher odds of DBM (pooled OR = 1.76, 95% CI: 1.20, 2.57; *I*^2^ = 77.5%) compared with vaginal delivery. Media exposure showed no significant association with DBM (pooled OR = 1.16; 95% CI: 0.79, 1.69; *I*^2^ = 66.4%).

### Additional factors associated with DBM

Beyond the 12 factors included in the meta-analysis, several additional determinants were identified across individual studies. Five studies [35,43,45,52,53] found that households where the child was a later-born child (second or higher in the birth order) had an increased risk of DBM compared with households with firstborn children. Similarly, 4 studies [34,38,43,52] reported that larger household size, measured by the total number of household members, was associated with higher DBM risk. Two studies [13,53] indicated that antenatal care (ANC) visits reduced DBM risk, whereas 4 studies [19,34,49,50] linked household food insecurity with higher DBM likelihood. Evidence from 1 study [44] suggested that higher protein intake among children and higher fat intake among mothers were associated with an increased risk of DBM.

### Subgroup analysis

**FIGURE 4:**
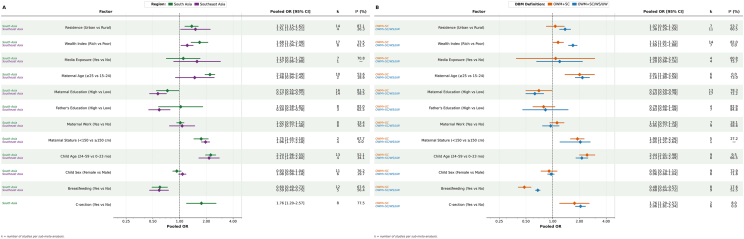
Subgroup analysis of factors associated with the DBM by region (A) and DBM definition (B). Last category of each factor is considered as reference category. K, number of studies to estimate the pooled estimate. CI, confidence interval; DBM, double burden of malnutrition; OWM + SC, overweight mother with stunted child; OWM + SC/WS/UW, overweight mother with stunted, wasted, or underweight child; OWM + UWC, overweight mother with underweight child; OWM + WC, overweight mother with wasted child.

To explore potential sources of heterogeneity and assess whether associations differed by DBM definition and region, we conducted subgroup analyses based on 2 commonly used DBM definitions (OWM + SC compared with composite definitions including OWM + SC/WS/UW) and geographic region (South Asia compared with Southeast Asia) (Figure 4).

Across DBM definitions, the direction and magnitude of associations were highly consistent (Figure 4). Older maternal age, higher household wealth, short maternal stature, older child age, and cesarean delivery were associated with increased likelihood of DBM under both definitions, with similar effect sizes (e.g., maternal age: OR = 2.01 compared with 2.16; child age: OR = 2.44 compared with 2.13). Likewise, maternal education and breastfeeding were protective under both definitions, with comparable effect estimates (maternal education: OR = 0.70 compared with 0.63; breastfeeding: OR = 0.48 compared with 0.68). These findings suggest that the associations between key determinants and DBM were robust and not meaningfully modified by DBM definition.

Similarly, regional subgroup analysis showed comparable effect sizes across South Asia and Southeast Asia (Figure 4). Higher wealth (OR = 1.68 compared with 1.55), short maternal stature (OR = 1.96 compared with 1.75), and older child age (OR = 2.24 compared with 2.15) were associated with increased likelihood of DBM in both regions. Maternal education (OR = 0.73 compared with 0.59) and breastfeeding (OR = 0.60 compared with 0.59) were protective in both regions. Although minor differences in magnitude were observed, the overall similarity in effect estimates indicates no clear evidence that geographical region substantially modified the associations between these factors and DBM. Subgroup analyses also reduced heterogeneity for several factors, suggesting that variation in DBM definitions and study settings contributed to between-study variability without fundamentally altering the observed relationships.

### Sensitivity analysis

**FIGURE 5:**
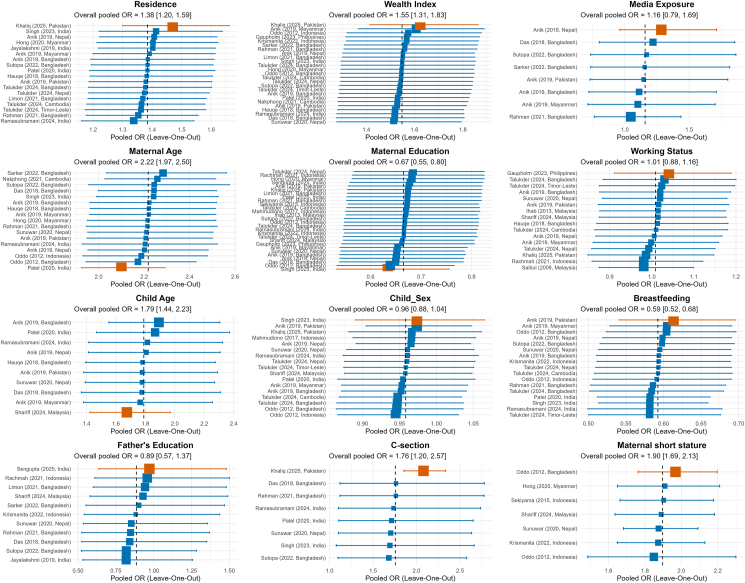
Sensitivity analysis showing the influence of individual studies on the pooled estimates of factors associated with DBM. Each data point represents the pooled OR recalculated after sequentially removing 1 study at a time. Blue squares represent individual leave-one-out pooled estimates with their 95% CIs. The orange square identifies the study whose removal produces the greatest change in the pooled estimate, indicating the single most influential study for that factor. The dashed vertical line represents the overall pooled estimate based on all included studies. CI, confidence interval; DBM, double burden of malnutrition; OR, odds ratio.

We conducted leave-one-out sensitivity analyses, removing 1 study at a time to assess the robustness of the pooled estimates (Figure 5). These analyses showed that the results for most factors were stable, suggesting that no single study had an undue influence. Urban residence, higher household wealth, older maternal age, maternal short stature, older child age, and cesarean delivery increased the risk of DBM across all iterations of the sensitivity analysis. On the other hand, maternal education and breastfeeding remained protective factors. However, a notable exception was observed for cesarean delivery, where the removal of the Khaliq et al.’s [38] study resulted in a meaningful upward shift in the pooled estimate, such that the lower bound of the 95% CI exceeded the original overall point estimate. This indicates that the Khaliq study exerts a moderating influence on the cesarean delivery estimate, and that the association between cesarean delivery and DBM may be stronger than the overall pooled estimate suggests when this study is excluded. This finding warrants cautious interpretation of the cesarean delivery result and highlights the need for additional studies on this association across diverse settings.

### Publication bias

**FIGURE 6:**
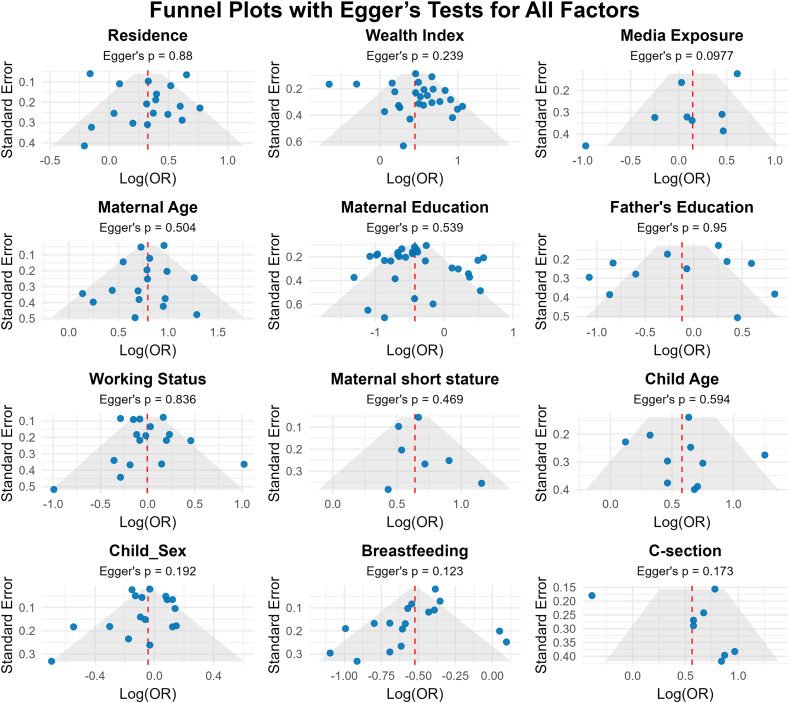
Funnel plots with results of Egger’s test assessing publication bias across all identified factors. OR, odds ratio.

The funnel plots presented in Figure 6 showed a largely symmetrical distribution, suggesting minimal publication bias across the included studies. This visual assessment was supported by Egger’s test, which yielded nonsignificant results (*P* > 0.05), further confirming that publication bias is unlikely to have influenced our findings.

## Discussion

This study addresses a critical evidence gap by providing the first comprehensive meta-analytic synthesis of household-level determinants of DBM across South and Southeast Asia. Although previous reviews have examined global prevalence and predictors without quantitative pooling, and a recent systematic review and meta-analysis was limited to a single-country context in Ethiopia, no prior study has generated region-specific pooled estimates for this diverse setting. By synthesizing evidence from 30 studies across this region and conducting meta-analysis for 12 factors, this review makes several contributions to existing literature. It provides the first region-specific pooled estimates for key risk factors, including urban residence, household wealth, maternal short stature, and notably, cesarean delivery, an association previously identified only in isolated studies. These findings challenge the common assumption that DBM is predominantly driven by poverty, underscore the importance of intergenerational influences, and highlight cesarean delivery as a potentially under-recognized upstream factor. Overall, this study advances the evidence base from fragmented single-country findings to a coherent, regionally grounded synthesis that can inform more targeted nutrition strategies in South and Southeast Asia.

### Household-level factors: urbanization, wealth, and nutrition transition

Our meta-analysis showed that both urban residence (38% higher likelihood) and higher household wealth (55% higher likelihood) were significantly associated with DBM, with both associations remaining robust across sensitivity analyses. These findings provide strong quantitative evidence that the burden is shifting toward more affluent and urban populations. This pattern is consistent with previous studies highlighting the role of rapid urbanization and economic growth in reshaping dietary patterns and lifestyles across the region [1,2,5,13,24,26]. In such environments, greater access to energy-dense, nutrient-poor foods and more sedentary lifestyles contribute to maternal overweight, whereas persistent structural and social barriers—such as limited access to diverse child foods, suboptimal feeding practices, and inadequate childcare—continue to drive child undernutrition [3,55].

The positive association between household wealth and DBM observed in our pooled estimates indicates that economic resources alone are insufficient to ensure optimal nutrition for all household members, a pattern also reported in Indonesia, Nepal, and Bangladesh [44,51,56]. Rather, these findings suggest that DBM is shaped by how resources are distributed and utilized within households. This may explain why DBM persists in wealthier settings, where maternal diets shift toward excess energy intake, whereas children continue to experience inadequate dietary diversity or care, reflecting intrahousehold nutritional inequalities [57]. At the same time, poorer households in urban areas may face disproportionate barriers to healthy diets despite living in food-rich environments. Structural constraints such as unhealthy food environments, limited income, and poor access to fresh foods may increase the risk of both undernutrition and overnutrition within the same household [58]. The stronger associations observed in South Asia compared with Southeast Asia in our subgroup analysis suggest that the mechanisms driving DBM may differ between regions. This could reflect more pronounced intrahousehold nutritional inequalities in South Asian contexts, potentially influenced by deeper gender disparities, differential feeding practices, or other sociocultural factors that shape resource allocation within households. It is important to note that we are measuring the coexistence of overnutrition and undernutrition within households, which may have distinct drivers beyond the nutrition transition factors that influence overnutrition alone.

### Maternal factors: the central role of mothers

Our meta-analysis identified older maternal age (≥25 y) as one of the strongest risk factors for household-level DBM, with these mothers having more than twice the likelihood of DBM compared with younger mothers. This large effect likely reflects 2 concurrent processes within the same household: older mothers are at higher risk of overweight due to cumulative weight retention from multiple pregnancies, metabolic changes, and reduced physical activity [59], whereas their children (often from earlier pregnancies) may experience persistent stunting due to suboptimal nutrition during critical growth periods [17].

Maternal short stature also emerged as a strong risk factor, associated with a 90% higher likelihood of DBM. Short stature is not merely a biological trait but serves as a marker of chronic undernutrition during the mother’s own childhood and adolescence [60,61]. This dual burden operates in 2 directions: short-statured mothers are more likely to have low-birth-weight infants and children with growth faltering [62,63], whereas simultaneously being at higher risk of overweight due to metabolic adaptations, lower lean body mass, and exposure to obesogenic environments [64]. Additionally, shorter individuals are more likely to be classified as overweight based on BMI, as BMI is a function of height and weight, making it easier for shorter people to reach the overweight threshold at lower absolute weights. These findings highlight the importance of improving adolescent nutrition as a strategy to prevent the intergenerational perpetuation of malnutrition.

In contrast, higher maternal education reduced the likelihood of DBM by 33%, making it one of the strongest modifiable protective factors. This protective effect is well established in the nutrition literature, as educated mothers generally possess better knowledge of nutrition, greater autonomy in household decision-making, and improved access to health services [13,21]. By enhancing mothers’ capacity to make informed feeding choices, access healthcare promptly, and support their children’s nutritional needs, education directly contributes to improved household nutrition [21]. In contrast, the absence of a significant association between paternal education and DBM suggests that mothers have a much stronger influence on household nutrition, given their central role in caregiving and food preparation in most South and Southeast Asian settings. Interestingly, maternal working status showed no significant association with DBM, which may be due to variations in employment types, working conditions, and the availability of childcare support across different settings.

### Child-level factors: age and developmental vulnerability

Children aged 24–59 mo were 79% more likely to be in a DBM-affected household compared with children aged 0–23 mo. This age gradient reflects the cumulative effects of inadequate complementary feeding, repeated infections, and environmental exposures during early childhood [65,66]. Growth faltering often accelerates after 6 mo of age, when breast milk alone becomes insufficient, and appropriate complementary foods are frequently unavailable or improperly introduced [67,68]. Meanwhile, maternal weight gain may continue or even accelerate during this period, particularly in households undergoing nutrition transition, further contributing to the household-level double burden.

### Behavioral and healthcare factors

Breastfeeding was associated with a 37% lower likelihood of household-level DBM, and this protective association was consistent across subgroups defined by both DBM definition and geographic region, making it one of the most robust findings of this review. This finding is consistent with extensive evidence on its benefits for both child growth and maternal health [69,70]. Exclusive breastfeeding for the first 6 mo, followed by continued breastfeeding ≤2 y or beyond, provides optimal nutrition for infants and young children while supporting maternal weight management through increased energy expenditure [23,71,72]. Additionally, breastfeeding reduces the risk of childhood infections, which are major contributors to stunting, further reinforcing its protective role [[73], [74], [75]]. These findings highlight the importance of promoting and supporting breastfeeding as a cornerstone of DBM prevention strategies.

Cesarean delivery was associated with a 76% higher likelihood of household-level DBM, although this estimate warrants cautious interpretation, as sensitivity analysis indicated that removing a single influential study increased the pooled estimate, suggesting that the true association may be even stronger than the overall result. Multiple plausible pathways may explain this association. Cesarean delivery can delay lactation initiation, reduce breastfeeding duration, and alter infant gut microbiota, all of which may negatively impact child growth [72,76,77]. Furthermore, cesarean delivery is more common among women with overweight or obesity, who may face higher risks of surgical complications and challenges in postpartum weight management [78,79]. Reduced mobility and physical activity during recovery may also exacerbate postpartum weight retention, contributing to maternal overweight in the household [79,80].

### Additional factors: emerging evidence

Several factors identified in the narrative synthesis, however not included in the meta-analysis due to limited data, are also important to consider. Higher birth order and larger household size were associated with increased DBM risk, likely due to reduced availability of food and caregiving for each child within the household [34,36,38,43,52]. Attending ANC visits was linked to reduced DBM risk, highlighting the importance of prenatal nutrition counseling and maternal health monitoring in mitigating household malnutrition [13,53]. Household food insecurity was also consistently associated with higher DBM likelihood, emphasizing the fundamental role of adequate food access in preventing malnutrition [19,34,49,50]. Moreover, several dietary factors—such as excessive protein intake in children, higher maternal fat intake, and imbalanced energy intake—may further contribute to DBM [34,44]. These findings indicate the need for longitudinal studies with detailed dietary assessments to clarify causal pathways and inform targeted dietary interventions.

### Implications for policy and practice

The findings of this systematic review challenge the longstanding assumption that malnutrition is primarily a problem of poverty and food insecurity [81]. DBM is increasingly concentrated in urban and wealthier households, meaning that conventional poverty-targeted nutrition programs are misaligned with the population now most affected [2,5]. National nutrition policies, therefore, need to expand beyond food security frameworks to include regulatory responses to obesogenic food environments, such as restrictions on unhealthy food marketing and fiscal policies on sugar-sweetened beverages [2]. At the same time, investment in child undernutrition programs must be sustained, as both forms of malnutrition coexist within the same households. Addressing the intergenerational nature of DBM is equally critical. Maternal short stature, older maternal age, and cesarean delivery all point to mechanisms that transmit nutritional risk across generations [4,62,63]. Investing in girls’ education and women’s empowerment directly disrupts these cycles by improving maternal nutrition knowledge, healthcare-seeking behavior, and household decision-making [13,21]. Programs targeting adolescent girls and young women before pregnancy can improve birth outcomes and reduce downstream DBM risk, and must therefore be repositioned as explicit upstream prevention strategies rather than standalone health activities [69,82]. Supporting breastfeeding through enforcement of the International Code of Marketing of Breast-milk Substitutes [82], paid maternity leave, and workplace lactation programs can simultaneously reduce child undernutrition and maternal overweight [72].

### Research recommendations

Our findings highlight the need for longitudinal studies with detailed dietary assessments to clarify the causal pathways underlying household-level DBM. Understanding these mechanisms is essential, as it allows interventions to address not only observed associations but also the processes through which maternal and child nutrition risks are transmitted across generations. Moreover, evidence on the long-term consequences and temporal dynamics of DBM remains limited, making prospective studies critical to track how undernutrition and overnutrition coevolve within households and across the life course [83]. In addition, future research should assess the impact of specific policies and large-scale nutrition programs, particularly those targeting obesity prevention, food environments, and social protection. Evidence from existing evaluations is limited and inconsistent, leaving gaps in understanding which interventions can simultaneously reduce both undernutrition and overweight [84]. Strengthening longitudinal research and conducting rigorous program evaluations will expand the evidence base needed to design and scale interventions that effectively reduce DBM in South and Southeast Asia.

### Strengths and limitations

This study represents one of the most comprehensive efforts to systematically review and quantitatively synthesize evidence on factors associated with household-level DBM in South and Southeast Asia. By integrating findings from multiple countries, it captures regional diversity and identifies both common and context-specific determinants of DBM. A comprehensive search across multiple databases, supplemented with manual searches, minimized the likelihood of missing relevant literature. All included studies demonstrated good methodological quality as assessed using the NOS-xs tool. Meta-analyses of 12 factors allowed quantification of pooled effects and assessment of heterogeneity, whereas subgroup analyses by DBM definition and region helped explain contextual variations. Sensitivity analyses and publication bias assessments confirmed the robustness and reliability of our findings, providing a strong evidence base to inform regional policy and programmatic responses.

However, several limitations should be acknowledged. First, all included studies employed cross-sectional designs, precluding causal inference. Longitudinal studies are needed to establish temporal relationships and identify critical windows for intervention. Second, substantial heterogeneity was observed for most factors, reflecting variations in study populations, DBM definitions, measurement methods, and analytical approaches. Although subgroup analyses partially explained this heterogeneity, residual variability remained. Third, the definition of DBM varied across studies, with some using composite definitions that combined multiple forms of child undernutrition. Although our subgroup analyses addressed this issue, standardized DBM definitions would facilitate more direct comparisons. Fourth, media exposure was consistently measured using a standard DHS definition (television, radio, or newspapers at least once per week). Although this supports comparability, it may not reflect current digital media landscapes, potentially limiting its validity in more recent study contexts. Fifth, we were unable to conduct meta-analyses for several potentially important factors due to insufficient data, including household size, birth order, food security, and dietary patterns. Sixth, a formal certainty of evidence assessment using the Grading of Recommendations, Assessment, Development and Evaluations approach was not undertaken, as its application to observational etiological evidence remains methodologically debated [85]. Readers should therefore interpret the pooled estimates with appropriate caution, considering the cross-sectional nature of included studies, the substantial heterogeneity observed for several factors, and the variation in DBM definitions across studies.

In conclusions, this systematic review and meta-analysis provide a comprehensive synthesis of household-level determinants of DBM in South and Southeast Asia. Findings of this study highlight the need for integrated, context-specific interventions that address both undernutrition and overnutrition across the life course. Policies should target not only poor and rural populations but also urban and affluent households experiencing nutrition transitions. Encouraging maternal education, promoting breastfeeding, and implementing interventions to prevent intergenerational malnutrition are critical to reducing DBM and achieving sustainable development goals related to nutrition and health. Future research should utilize longitudinal designs to establish causality, standardize DBM definitions to enhance comparability, and investigate the role of dietary patterns, food security, and household dynamics in DBM etiology.

## Author contributions

The authors’ responsibilities were as follows – AT, HS: conceptualized and designed the study; AT, MAS: conducted the literature search; HS: resolved any discrepancies in the selected articles; AT: led the data analysis and interpretation of the results and drafted the manuscript; HS, MK, DG: supervising the analysis output; HS, MK, MAS, DG: critically revised the manuscript for intellectual content; and all authors: reviewed and edited subsequent drafts, read and approved the final version, and contributed to the decision to submit for publication.

## Data availability

This review relied exclusively on publicly accessible data sources. The data processing methods described in the manuscript, along with the R scripts, are available from the corresponding author upon reasonable request.

## Funding

The authors reported no funding received for this study.

## Declaration of Generative AI and AI-assisted technologies in the writing process

The author(s) declare that no generative AI or AI-assisted technologies were used in the writing of this manuscript.

## Conflict of interest

The authors report no conflicts of interest.
