# Supplementary material for: Determinants of Household-Level Double Burden of Malnutrition in South and Southeast Asia: A Systematic Review and Meta-analysis
Source: Curr Dev Nutr. 2026 Apr 1;10(5):107683. doi: 10.1016/j.cdnut.2026.107683 (PMC13123343; doi:10.1016/j.cdnut.2026.107683)
Supplement: multimedia component 1 [file mmc1.pdf]

Supplementary table S1: Search strategy

Source	Search strategy
**PubMed (Total search: 237)**	("double burden"[all] OR double burden[all] OR "dual burden"[all] OR coexist*[all] OR concurrent[all] OR (overweight[all] AND (undernutri*[all] OR underweight[all] OR stunt*[all] OR wast*[all]) AND (double[all] OR dual[all] OR coexist*[all] OR concurrent[all])))<br>AND<br>(Mother [Mesh] OR Child [Mesh] OR Parents [Mesh] OR household [all] OR households [all] OR mother[all] OR maternal[all] OR mother-child[all] OR "mother and child"[all] OR dyad[all] OR pair[all] OR parent*[all] OR caregiver[all])<br>AND<br>(Malnutrition [Mesh] OR Overweight [Mesh] OR Obesity [Mesh] OR Underweight [Mesh] OR "Protein-Energy Malnutrition"[Mesh] OR "Growth Disorders"[Mesh])<br>AND<br>(factor*[all] OR determinant*[all] OR associated[all] OR association[all] OR predictor*[all] OR correlate*[all] OR "risk factor*"[all])<br>AND<br>(Afghanistan[all] OR Bangladesh[all] OR Bhutan[all] OR India[all] OR Maldives[all] OR Nepal[all] OR Pakistan[all] OR "Sri Lanka"[all] OR Brunei[all] OR Cambodia[all] OR Indonesia[all] OR Laos[all] OR "Lao PDR"[all] OR Malaysia[all] OR Myanmar[all] OR Philippines[all] OR Singapore[all] OR Thailand[all] OR Timor[all] OR "East Timor"[all] OR Timor-Leste[all] OR Vietnam[all])
**Scopus (Total search: 367)**	TITLE-ABS-KEY (("double burden" OR double-burden OR "dual burden" OR coexist* OR concurrent OR (overweight AND (undernutri* OR underweight OR stunt* OR wast*) AND (double OR dual OR coexist* OR concurrent)))<br>AND<br>(Mother OR Child OR Parents OR household OR households OR mother OR maternal OR mother-child OR "mother and child" OR dyad OR pair OR parent* OR caregiver)<br>AND<br>(Malnutrition OR Overweight OR Obesity OR Underweight OR "Protein-Energy Malnutrition" OR "Growth Disorders")<br>AND<br>(factor* OR determinant* OR associated OR association OR predictor* OR correlate* OR "risk factor*")<br>AND<br>(Afghanistan OR Bangladesh OR Bhutan OR India OR Maldives OR Nepal OR Pakistan OR "Sri Lanka" OR Brunei OR Cambodia OR Indonesia OR Laos OR "Lao PDR" OR Malaysia OR Myanmar OR Philippines OR Singapore OR Thailand OR Timor OR "East Timor" OR "Timor-Leste" OR Vietnam)

	)
**Web of science (Total search: 417)**	("double burden" OR "double burden" OR "dual burden" OR coexist* OR concurrent OR (overweight AND (undernutri* OR underweight OR stunt* OR wast*) AND (double OR dual OR coexist* OR concurrent)))<br>AND<br>(Mother OR Child OR Parents OR household OR households OR mother OR maternal OR mother-child OR "mother and child" OR dyad OR pair OR parent* OR caregiver)<br>AND<br>(Malnutrition OR Overweight OR Obesity OR Underweight OR "Protein-Energy Malnutrition" OR "Growth Disorders")<br>AND<br>(factor* OR determinant* OR associated OR association OR predictor* OR correlate* OR "risk factor*")<br>AND<br>(Afghanistan OR Bangladesh OR Bhutan OR India OR Maldives OR Nepal OR Pakistan OR "Sri Lanka" OR Brunei OR Cambodia OR Indonesia OR Laos OR "Lao PDR" OR Malaysia OR Myanmar OR Philippines OR Singapore OR Thailand OR Timor OR "East Timor" OR Timor-Leste OR Vietnam)

Title: Determinants of Household-Level Double Burden of Malnutrition in South and Southeast Asia: A Systematic Review and Meta-Analysis

1st Author: Ashis Talukder

Supplementary Table S2. Characteristics of studies included in the systematic review on household-level DBM in South and Southeast Asia

Author	Country	Sample Size	DBM definition	Effect Measures	Study design	Suitable for meta	NOS-xs Score
Anik et al. (2019) [26]	Bangladesh, Nepal, Pakistan, Mayanmar	Bangladesh: 6,478 Nepal: 2,670 Pakistan: 5,770 Myanmar: 3,541	OWM+SC	AOR	Cross sectional	Yes	9
Agdeppa et al. (2003) [31]	Philippines	376 mother-child pairs	OWM+UW	Descriptive Statistics only	Cross sectional	No	7
Biswas et al. (2020) [24]	8 South and Southeast Asian Countries	798,961 households	OWM+SC, OWM+WC, OWM+UWC, OWM+SC/WC/UW	OR	Cross sectional	No	9
Das et al. (2018) [32]	Bangladesh	5951 households	OWM+SC, OWM+WC, OWM+UWC, OWM+SC/WC/UW	OR	Cross sectional	Yes	9
Fooken et al (2021)[33]	11 Asian Countries	134,283 households	OWM+SC	Descriptive Statistics only	Cross sectional	No	9
Gaupholm et al. (2023) [34]	Philippines	5837 households	OWM+SC/WC/UW	AOR	Cross sectional	Yes	9
Hauqe et al. (2017) [35]	Bangladesh	5697 mother-child pairs	OWM+SC/WC/UW	RR	Cross sectional	Yes	9
Hong et al. (2020) [23]	Myanmar	5687 mother-child pairs	OWM+SC	AOR	Cross sectional	Yes	9
Ihab et al. (2013) [36]	Malaysia	223 households	OWM+SC/WC/UW	AOR	Cross sectional	Yes	8
Jayalakshmi et al. (2019) [37]	India	344 mother and child pairs	OWM+SC	Descriptive Statistics only	Cross sectional	Yes	8
Khaliq et al. (2025) [38]	Pakistan	6198 mother-child pairs	OWM+SC	AOR	Cross sectional	Yes	9
Khor et al. (2003) [39]	Malaysia	140 households	OWM+UW	Descriptive Statistics only	Cross sectional	No	7
Krismanita et al. (2022) [40]	Indonesia	274 households	OWM+SC	OR	Cross sectional	Yes	8
Limon et al. (2021) [41]	Bangladesh	3772 mother-child pairs	OWM+SC/WC/UW	AOR	Cross sectional	Yes	8
Mahmudiono et al. (2018) [54]	Indonesia	685 households	OWM+SC	OR	Cross sectional	Yes	9

Nakphong et al. (2021) [42]	Cambodia	14,988 mother-child pairs	OWM+SC	AOR	Cross sectional	Yes	8
Oddo et al. (2012) [43]	Indonesia, Bangladesh	Indonesia: 247126 Bangladesh: 168317	OWM+SC	AOR	Cross sectional	Yes	8
Patel et al. (2020) [25]	India	184,680 mother-child pairs	OWM+SC/WC/UW	AOR	Cross sectional	Yes	9
Rachmah et al. (2021) [44]	Indonesia	436 (households)	OWM+SC	OR	Cross sectional	Yes	8
Rahman et al. (2021) [45]	Bangladesh	7,662 mother-child pairs	OWM+SC, OWM+WC, OWM+UWC, OWM+SC/WC/UW	AOR	Cross sectional	Yes	9
Ramasubramani et al. (2024)[10]	India	167,380 mother-child pairs	OWM+SC/WC	AOR	Cross sectional	Yes	9
Saibul et al. (2009) [46]	Malaysia	227 households	OWM+SC/WC/UW	AOR	Cross sectional	Yes	7
Sarker et al. (2022) [47]	Bangladesh	8,697 mother-child pairs	OWM+SC/WC/UW	AOR	Cross sectional	Yes	9
Sekiyama et al. (2015) [48]	Indonesia	242 mother-child pairs	OWM+SC	OR	Cross sectional	Yes	8
Sengupta et al. (2025) [49]	India	360 households	OWM+SC/WC/UW	AOR	Cross sectional	Yes	8
Shariff et al. (2024) [50]	Malaysia	451 households	OWM+SC/UW	AOR	Cross sectional	Yes	9
Singh et al. (2023)[52]	India	122,922 mother-child pairs	OWM+SC/WC/UW	AOR	Cross sectional	Yes	9
Sunuwar et al. (2020) [51]	Nepal	2261 mother-child pairs	OWM+SC, OWM+WC, OWM+UWC	AOR	Cross sectional	Yes	9
Sutopa et al. (2022) [53]	Bangladesh	14,975 mother-child pairs	OWM+SC/WC/UW	AOR	Cross sectional	Yes	9
Talukder et al. (2024) [13]	Bangladesh, Cambodia, Nepal, Timor-Leste	Total: 18,459 Bangladesh: 7,718 Cambodia: 3,836 Nepal: 2,596 Timor-Leste: 6,309	OWM+SC/WC/UW	AOR	Cross sectional	Yes	9

Note: AOR= adjusted odds ratios; **OR**= odds ratios; **OWM+SC**= overweight mother–stunted child; **OWM+WC**=overweight mother–wasted child; **OWM+UWC** overweight mother–underweight child; **OWM+SC/WC/UW**=overweight mother with any stunted, wasted, or underweight child; **NOS-xs**= Newcastle–Ottawa Scale adapted for cross-sectional studies.

Supplementary Table S3

Newcastle–Ottawa Scale adapted for cross-sectional studies (NOS-xs) quality assessment of included studies

Author	Country	Domain 1: Study Sample Selection (Max: 2 ★)		Domain 2: Assessment of Exposure and Outcome (Max: 4 ★)				Domain 3: Confounding Factors (Max: 3 ★)			Total Score (Max: 9)	Quality Rating
		Representativeness of Sample	Adequacy of Sample Size	Validity of Exposure Measurement	Reliability of Exposure Measurement	Validity of Outcome Measurement	Reliability of Outcome Measurement	Appropriate Control of Confounders	Measurement of Confounders	Reporting of Confounders		
Anik et al. (2019)	Bangladesh, Nepal, Pakistan, Myanmar	★	★	★	★	★	★	★	★	★	9/9	Low Risk (Good)
Agdeppa et al. (2003)	Philippines	★	—	★	★	★	★	★	—	★	7/9	Low Risk (Good)
Biswas et al. (2020)	8 South & Southeast Asian Countries	★	★	★	★	★	★	★	★	★	9/9	Low Risk (Good)
Das et al. (2018)	Bangladesh	★	★	★	★	★	★	★	★	★	9/9	Low Risk (Good)
Fooken et al. (2021)	11 Asian Countries	★	★	★	★	★	★	★	★	★	9/9	Low Risk (Good)
Gaupholm et al. (2023)	Philippines	★	★	★	★	★	★	★	★	★	9/9	Low Risk (Good)
Hauqe et al. (2018)	Bangladesh	★	★	★	★	★	★	★	★	★	9/9	Low Risk (Good)
Hong et al. (2020)	Myanmar	★	★	★	★	★	★	★	★	★	9/9	Low Risk (Good)
Ihab et al. (2013)	Malaysia	★	★	★	★	★	★	★	—	★	8/9	Low Risk (Good)
Jayalakshmi et al. (2019)	India	★	★	★	★	★	★	★	—	★	8/9	Low Risk (Good)
Khaliq et al. (2025)	Pakistan	★	★	★	★	★	★	★	★	★	9/9	Low Risk (Good)
Khor et al. (2003)	Malaysia	★	—	★	★	—	★	★	★	★	7/9	Low Risk (Good)
Krismanita et al. (2022)	Indonesia	★	★	★	★	★	★	★	—	★	8/9	Low Risk (Good)
Limon et al. (2021)	Bangladesh	★	★	★	★	★	★	★	—	★	8/9	Low Risk (Good)
Mahmudiono et al. (2018)	Indonesia	★	★	★	★	★	★	★	★	★	9/9	Low Risk (Good)
Nakphong et al. (2021)	Cambodia	★	★	★	★	★	★	★	—	★	8/9	Low Risk (Good)
Oddo et al. (2012)	Indonesia, Bangladesh	★	★	★	★	★	★	★	—	★	8/9	Low Risk (Good)
Patel et al. (2020)	India	★	★	★	★	★	★	★	★	★	9/9	Low Risk (Good)
Rachmah et al. (2021)	Indonesia	★	★	★	★	★	★	★	—	★	8/9	Low Risk (Good)
Rahman et al. (2021)	Bangladesh	★	★	★	★	★	★	★	★	★	9/9	Low Risk (Good)
Ramasubramani et al. (2024)	India	★	★	★	★	★	★	★	★	★	9/9	Low Risk (Good)
Saibul et al. (2009)	Malaysia	★	—	★	★	★	★	—	★	★	7/9	Low Risk (Good)
Sarker et al. (2022)	Bangladesh	★	★	★	★	★	★	★	★	★	9/9	Low Risk (Good)

Author	Country	Domain 1: Study Sample Selection (Max: 2 ★)		Domain 2: Assessment of Exposure and Outcome (Max: 4 ★)				Domain 3: Confounding Factors (Max: 3 ★)			Total Score (Max: 9)	Quality Rating
		Representativeness of Sample	Adequacy of Sample Size	Validity of Exposure Measurement	Reliability of Exposure Measurement	Validity of Outcome Measurement	Reliability of Outcome Measurement	Appropriate Control of Confounders	Measurement of Confounders	Reporting of Confounders		
Sekiyama et al. (2015)	Indonesia	★	★	★	★	★	★	★	—	★	8/9	Low Risk (Good)
Sengupta et al. (2025)	India	★	★	★	★	★	★	★	—	★	8/9	Low Risk (Good)
Shariff et al. (2024)	Malaysia	★	★	★	★	★	★	★	★	★	9/9	Low Risk (Good)
Singh et al. (2023)	India	★	★	★	★	★	★	★	★	★	9/9	Low Risk (Good)
Sunuwar et al. (2020)	Nepal	★	★	★	★	★	★	★	★	★	9/9	Low Risk (Good)
Sutopa et al. (2022)	Bangladesh	★	★	★	★	★	★	★	★	★	9/9	Low Risk (Good)
Talukder et al. (2024)	Bangladesh, Cambodia, Nepal, Timor-Leste	★	★	★	★	★	★	★	★	★	9/9	Low Risk (Good)

★ = Criterion met (1 star) | — = Criterion not met (0 stars)

Quality rating thresholds: Low Risk / Good Quality: 7–9 stars | Moderate Risk: 5–6 stars | High Risk: 0–4 stars

Scale: Newcastle–Ottawa Scale adapted for cross-sectional studies (NOS-xs). Carra et al., J Periodontal Res, 2025.

Figures: SF1-SF12: Forrest plots of factors associated with double burden of malnutrition in South and Southeast Asia

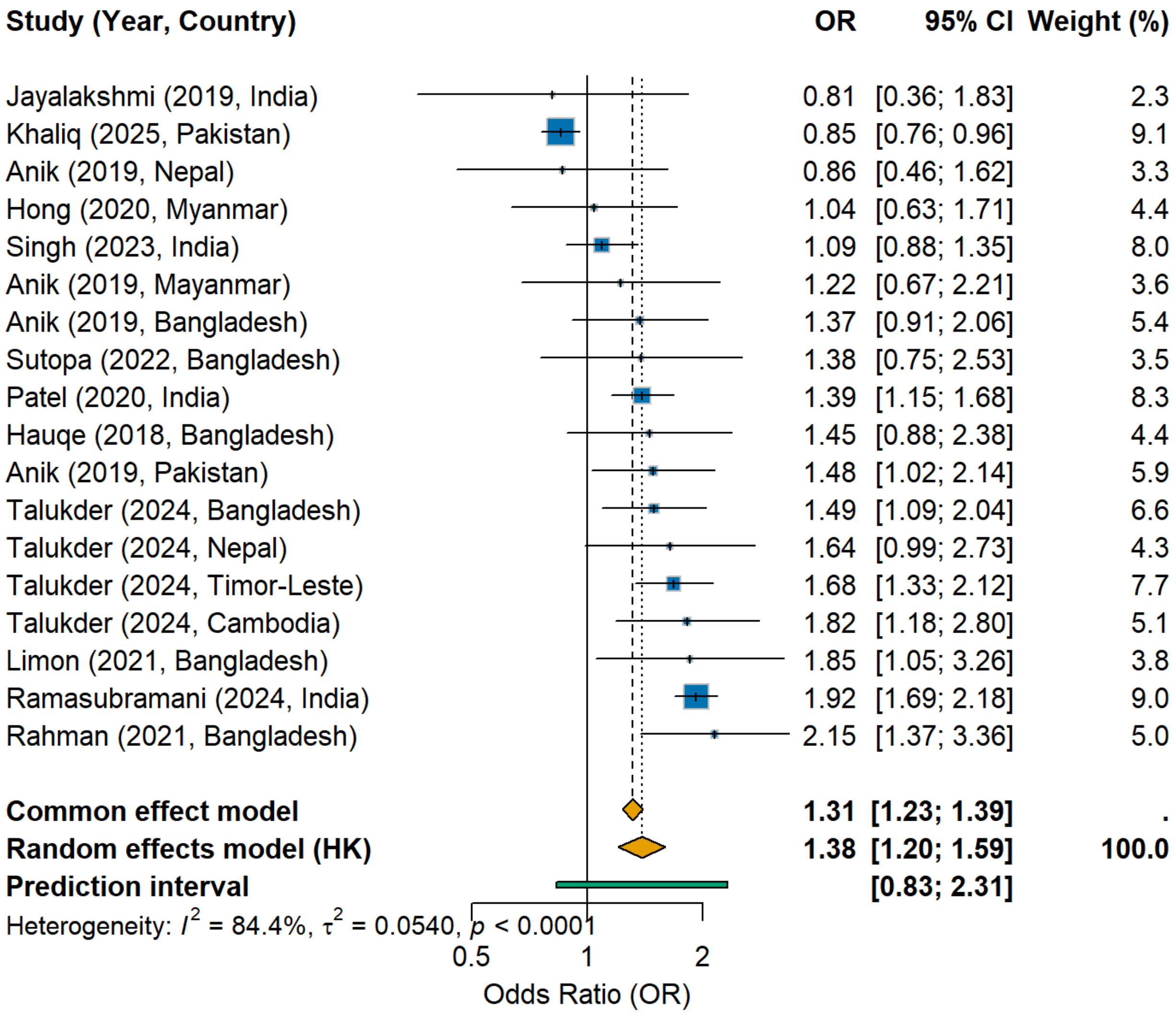

Figure: SF1: Forest plot for factor: Residence (Urban vs Rural)

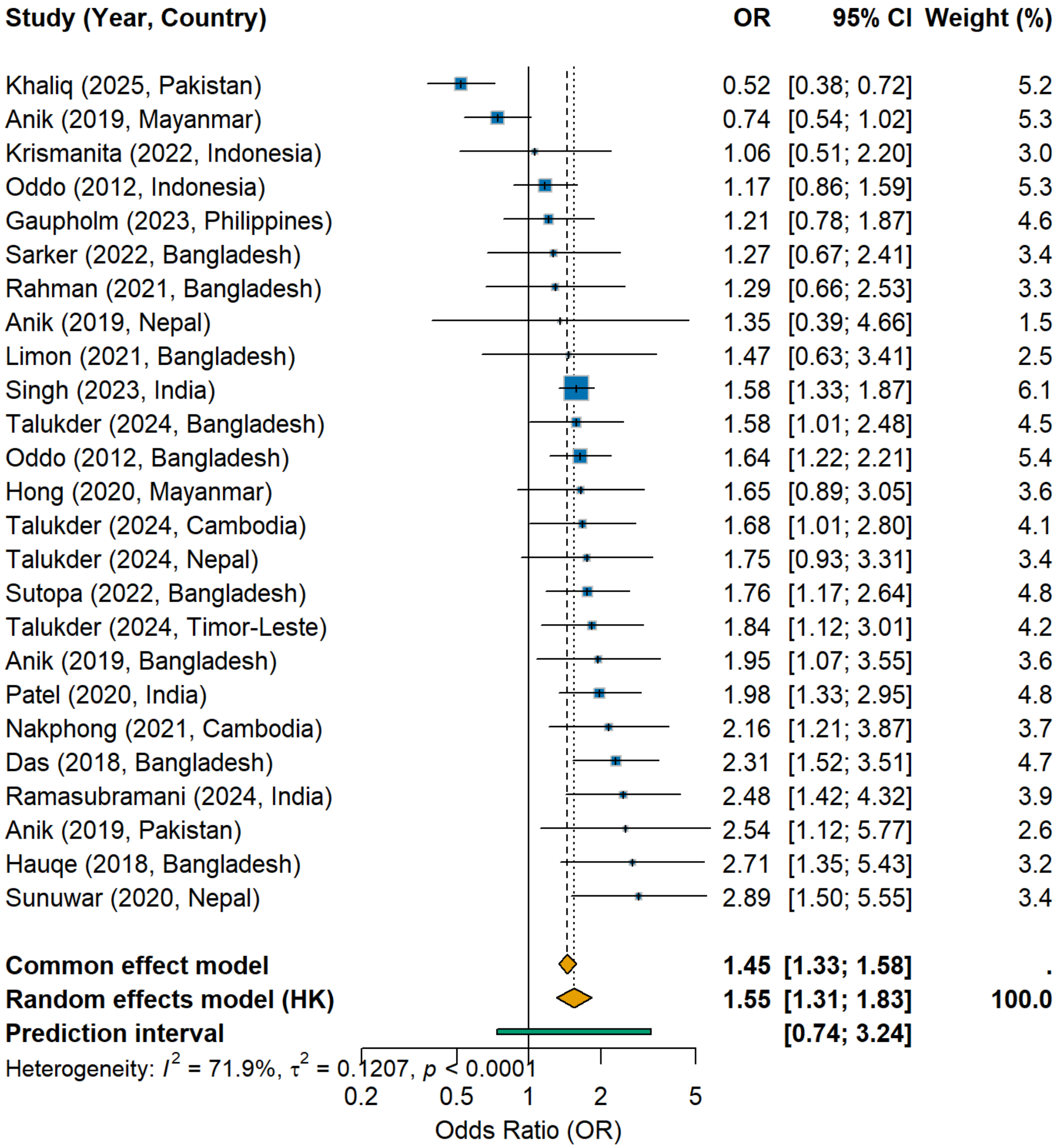

Figure: SF2: Forest plot for factor: Wealth Index (Rich vs Poor)

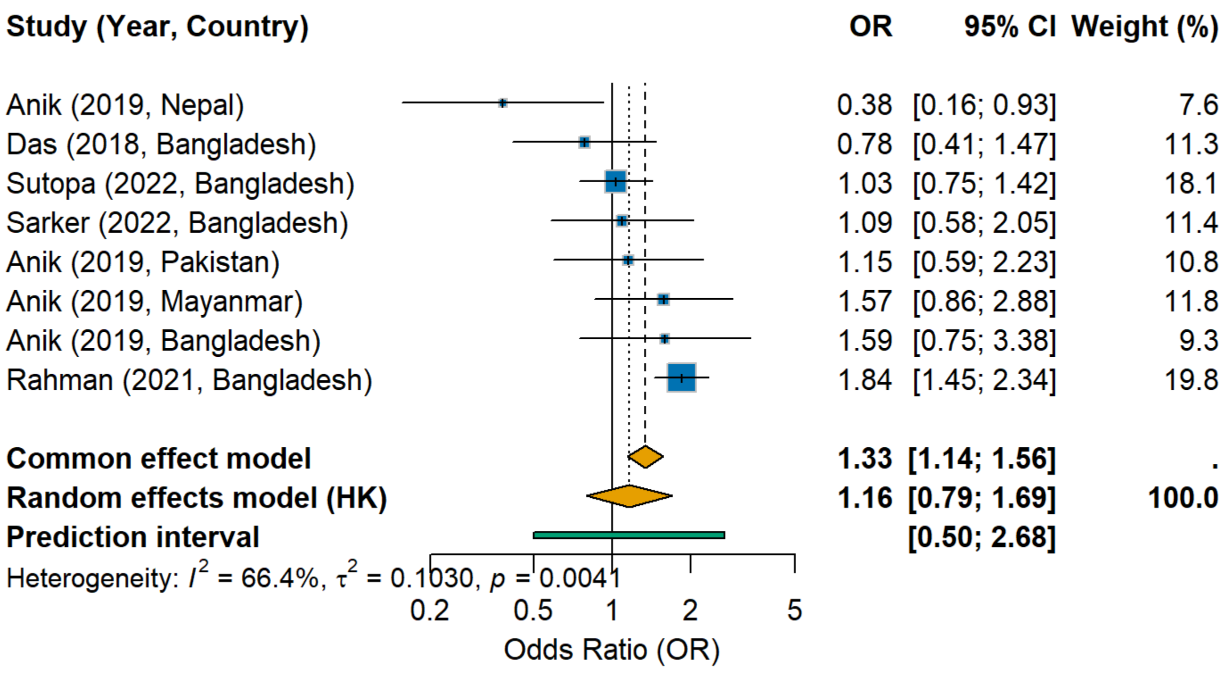

Figure: SF3: Forest plot for factor: Media Exposure (Yes vs No)

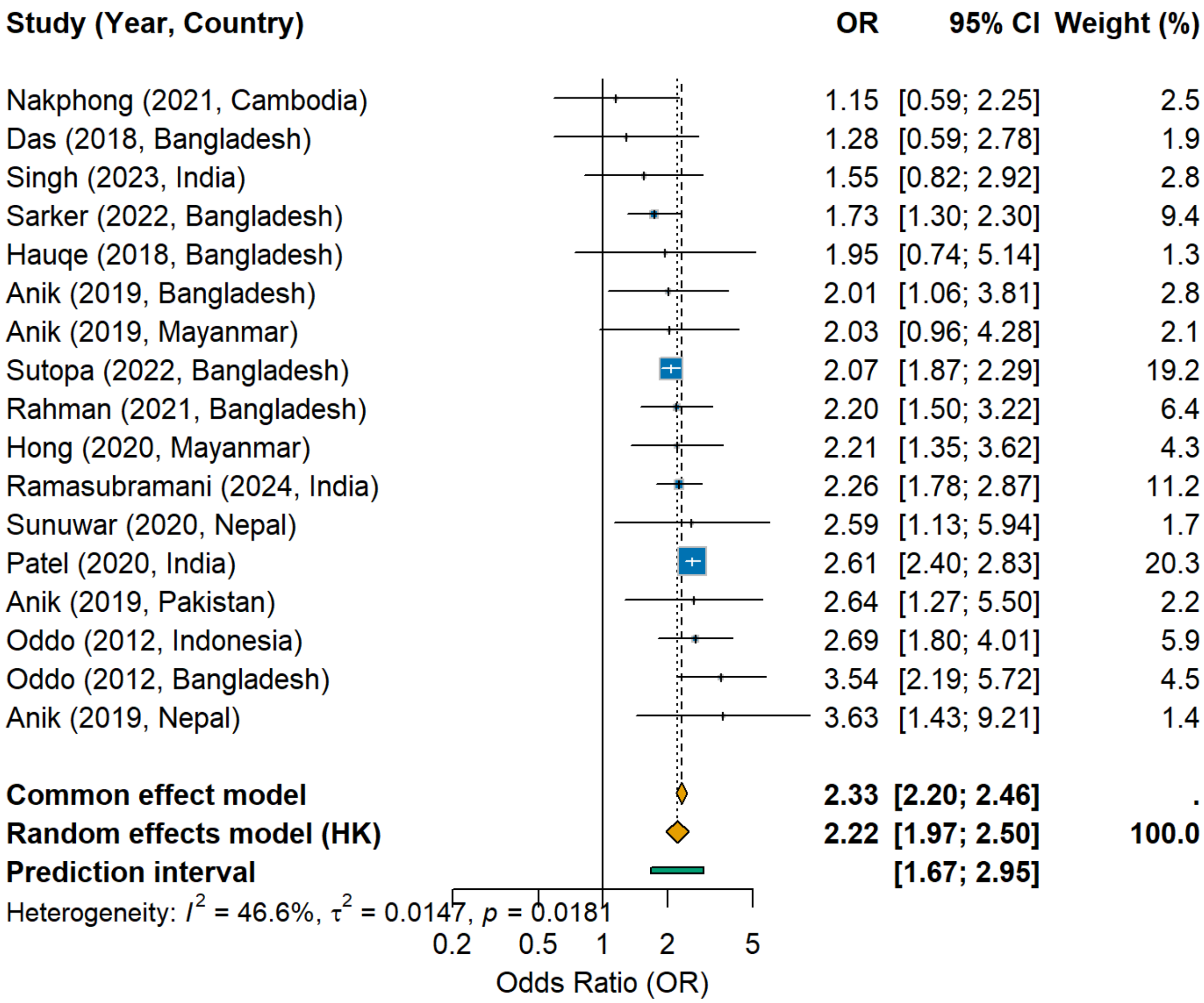

Figure: SF4: Forest plot for factor: Maternal Age (≥25 vs vs 15-24 years)

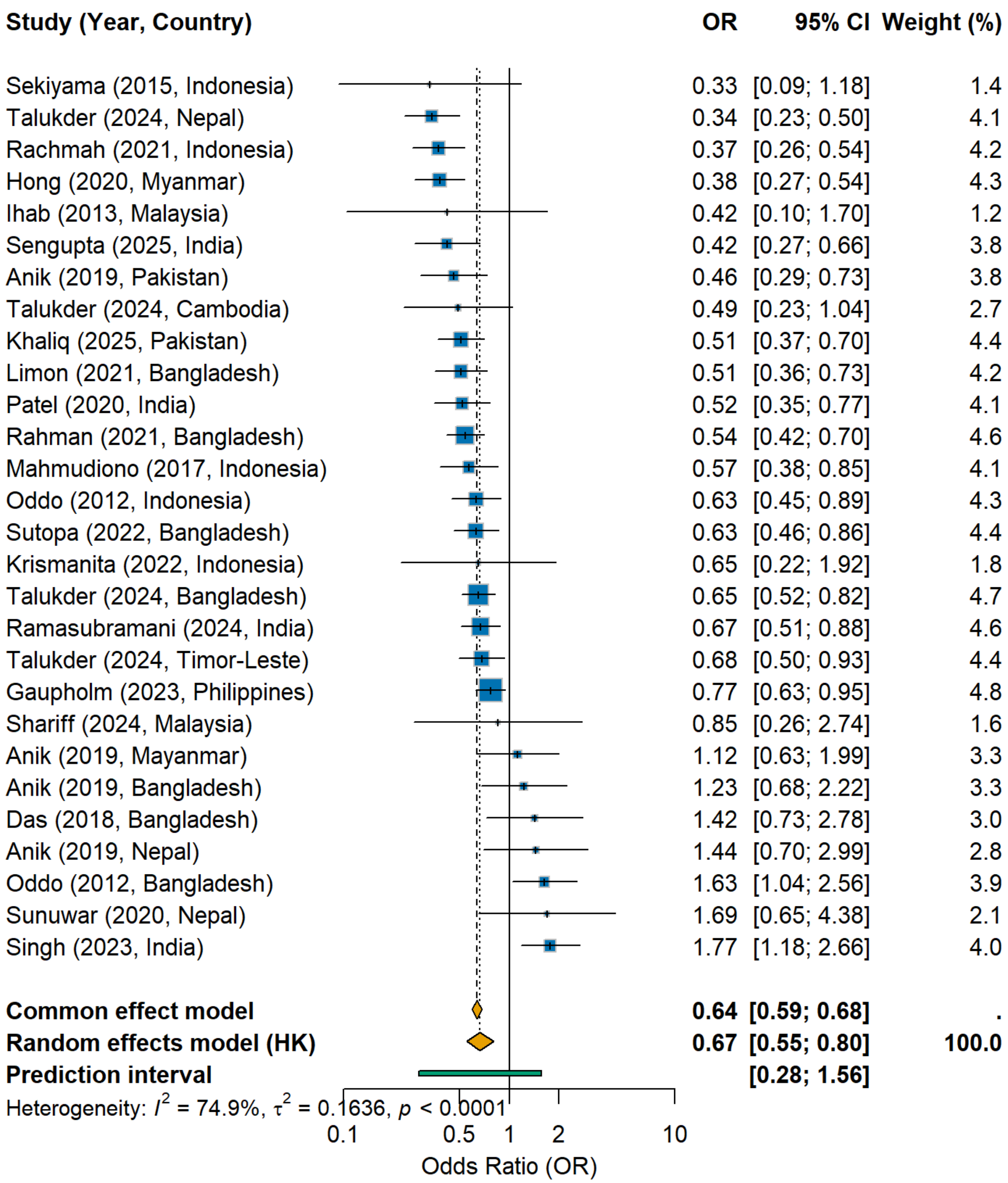

Figure: SF5: Forest plot for factor: Maternal Education (High vs Low)

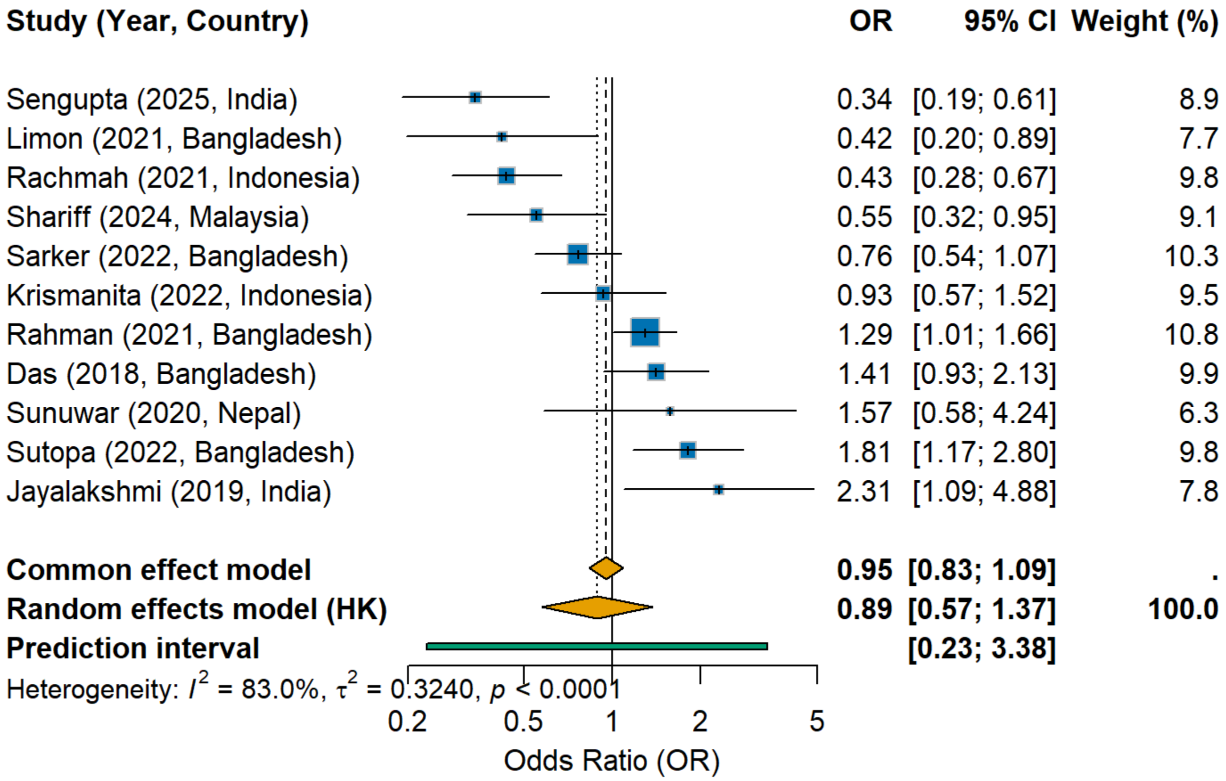

Figure: SF6: Forest plot for factor: Father's Education (High vs Low)

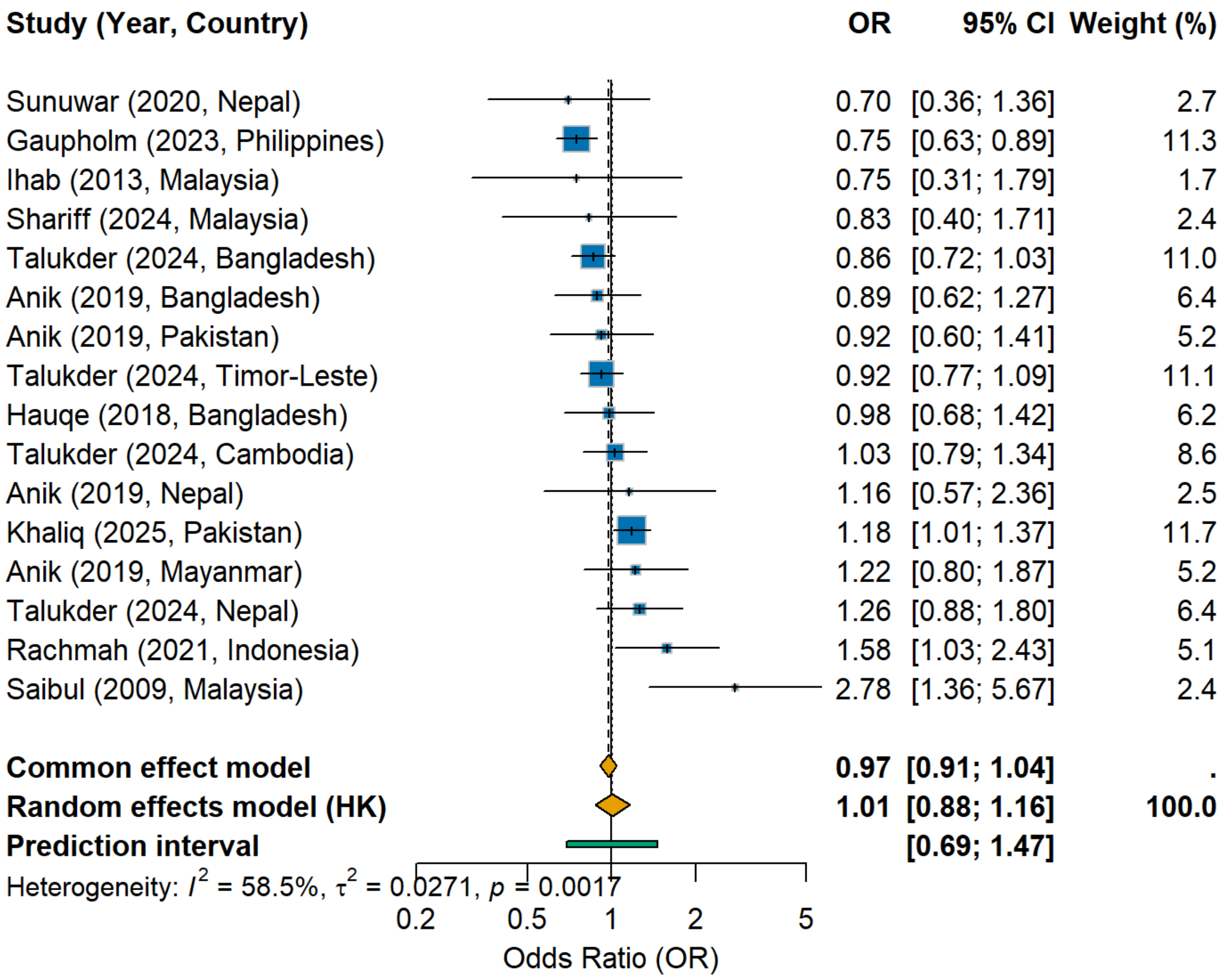

Figure: SF7: Forest plot for factor: Maternal Working Status (Yes vs No)

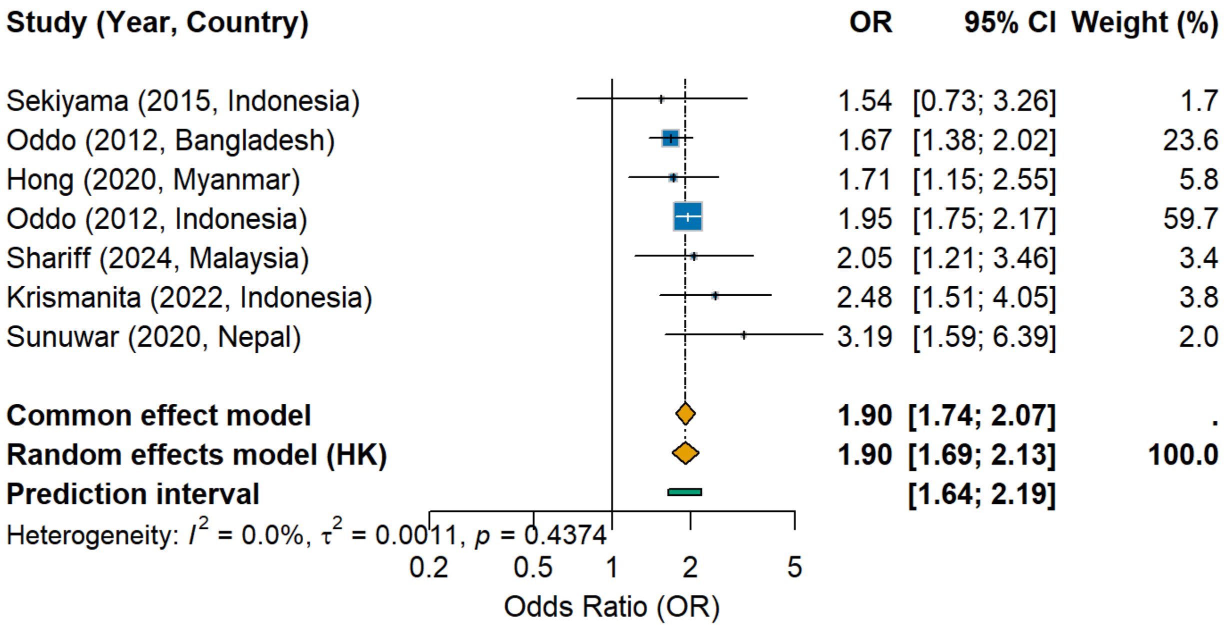

Figure: SF8: Forest plot for factor: Maternal Stature (<150 vs≥150 cm)

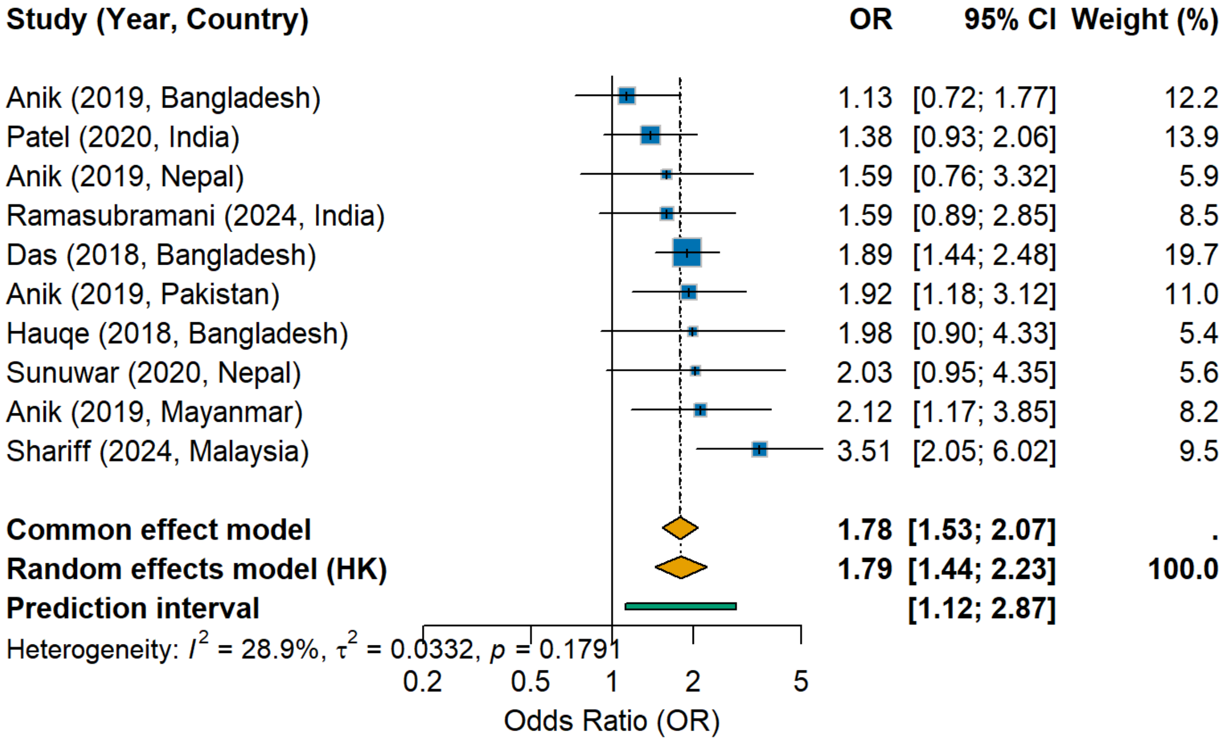

Figure: SF9: Forest plot for factor: Child Age (24-59 vs 0-23 months)

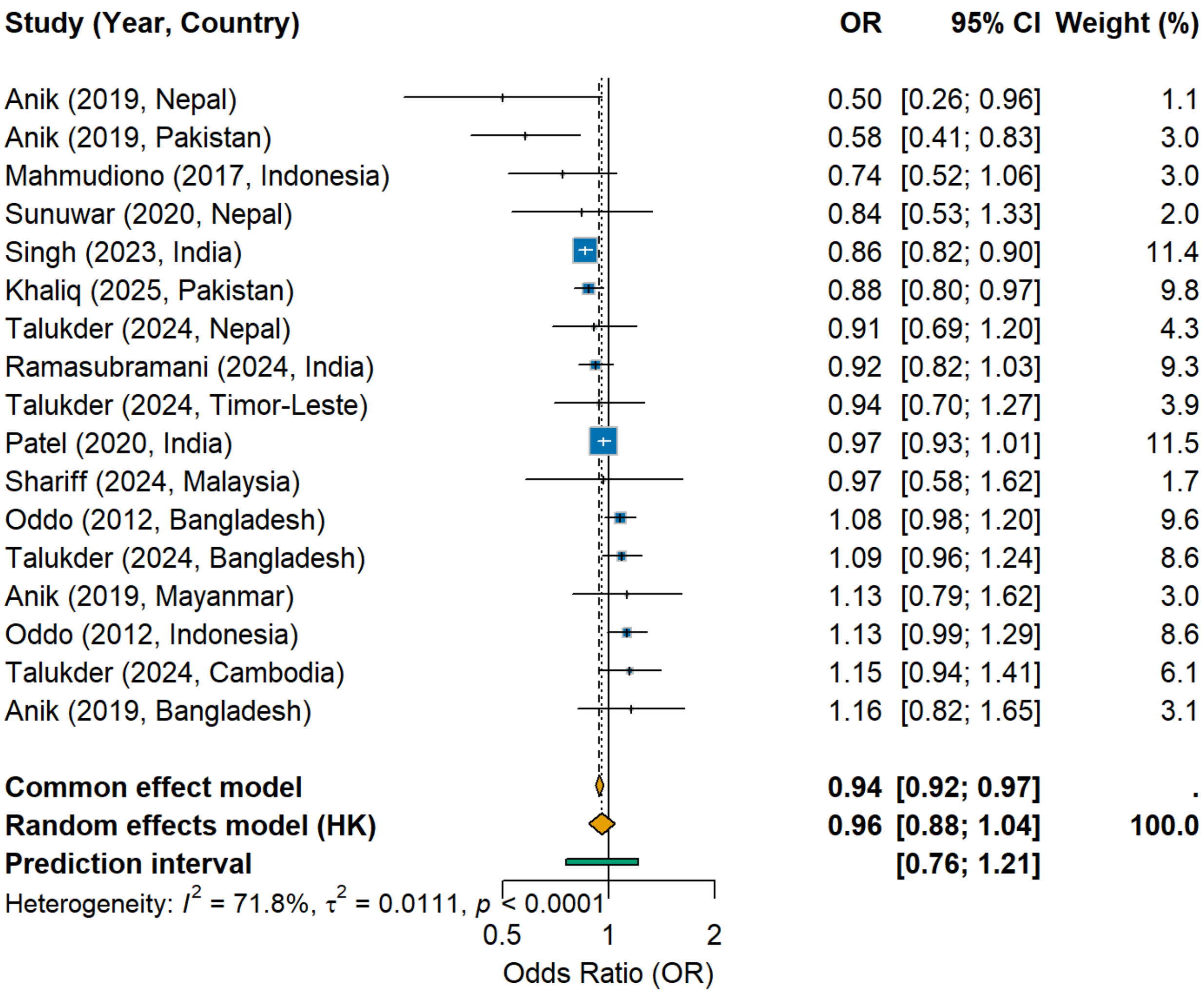

Figure: SF10: Forest plot for factor: Child Sex (Female vs Male)

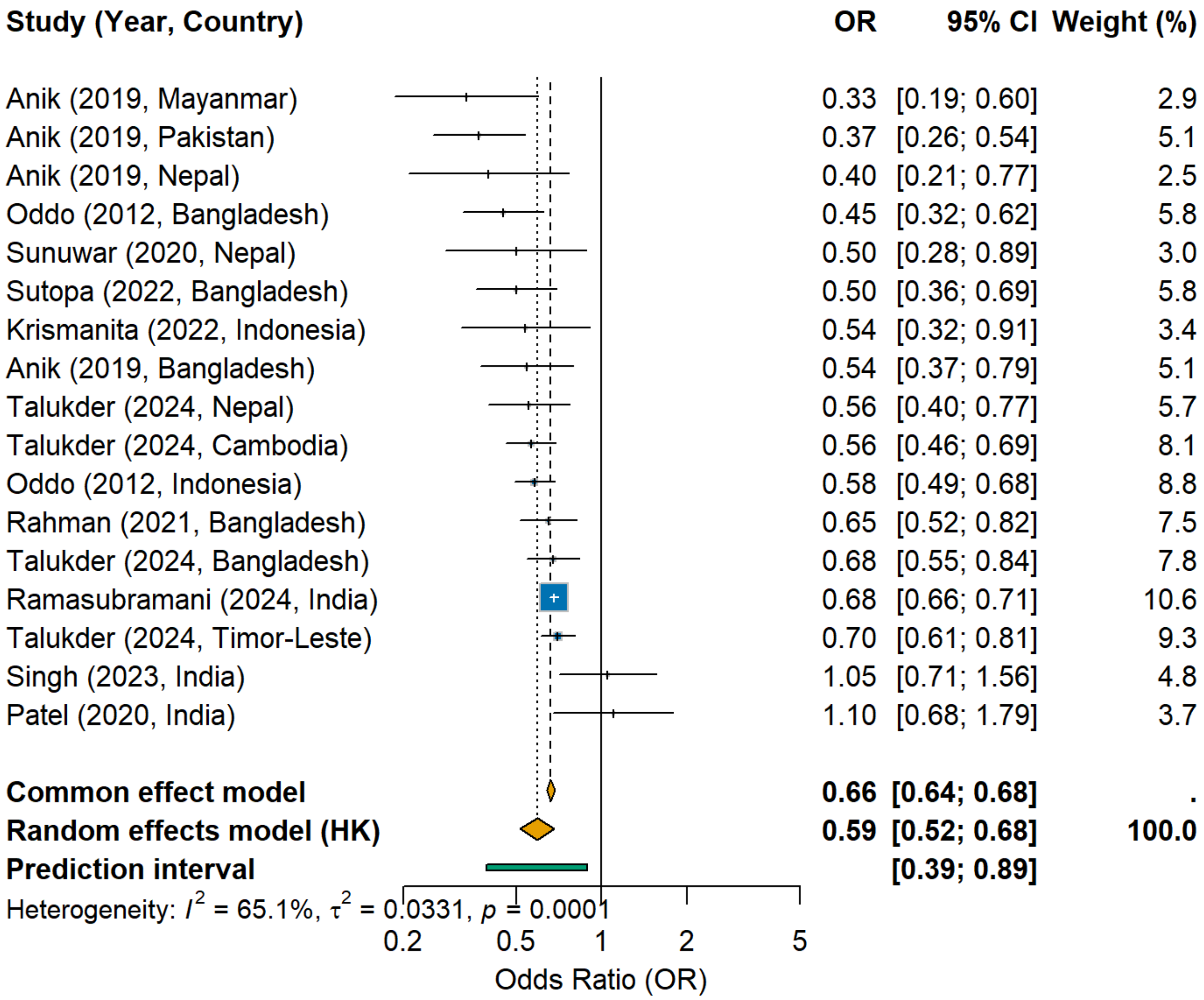

Figure: SF11: Forest plot for factor: Breastfeeding (Yes vs No)

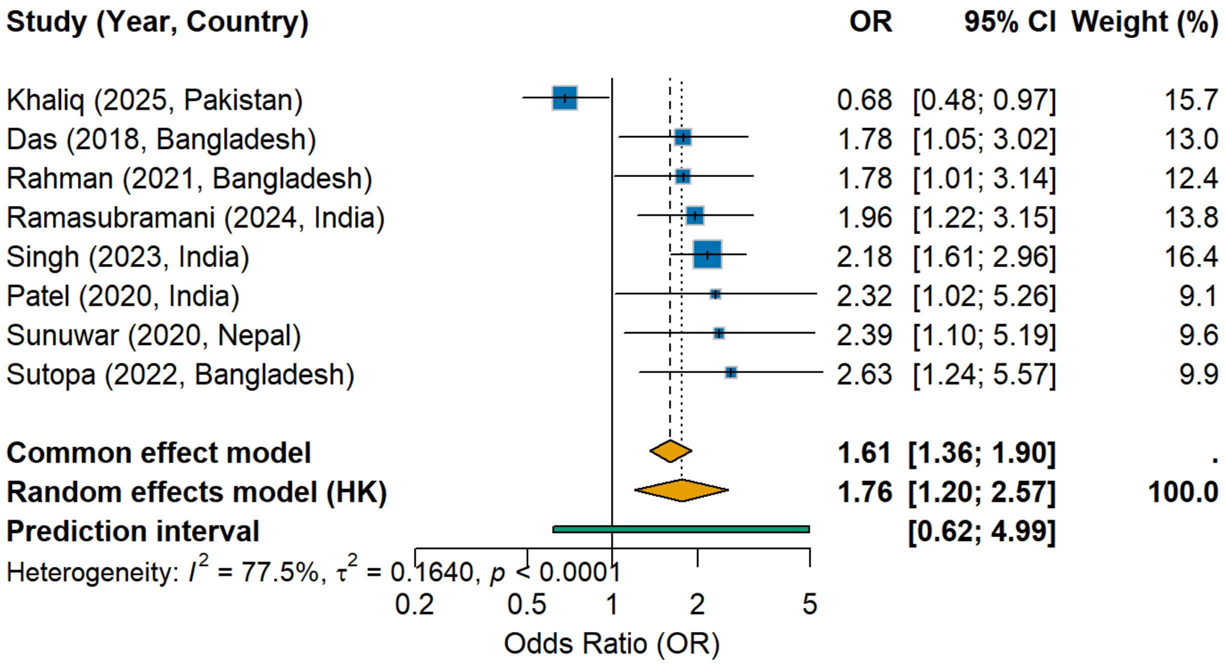

Figure: SF12: Forest plot for factor: C-section (Yes vs No)

PRISMA 2020 Main Checklist

Topic	No.	Item	Location where item is reported
**TITLE**			
**Title**	1	Identify the report as a systematic review.	Page 1, Lines 1–2 (Title)
**ABSTRACT**			
**Abstract**	2	See the PRISMA 2020 for Abstracts checklist	Page 2, Lines 29–53 (Abstract section)
**INTRODUCTION**			
**Rationale**	3	Describe the rationale for the review in the context of existing knowledge.	Pages 5–6, Lines 147–197 (Introduction section)
**Objectives**	4	Provide an explicit statement of the objective(s) or question(s) the review addresses.	Page 6, Lines 179–197 (Introduction, last paragraph)
**METHODS**			
**Eligibility criteria**	5	Specify the inclusion and exclusion criteria for the review and how studies were grouped for the syntheses.	Page 6, Lines 204–216 (Methods: Study selection and eligibility criteria; Exclusion Criteria)
**Information sources**	6	Specify all databases, registers, websites, organisations, reference lists and other sources searched or consulted to identify studies. Specify the date when each source was last searched or consulted.	Pages 6–7, Lines 217–225 (Methods: Search Strategy)
**Search strategy**	7	Present the full search strategies for all databases, registers and websites, including any filters and limits used.	Pages 6–7, Lines 217–225 (Methods: Search Strategy); Page 9, Line 290 (Results: Overview of literature screening)
**Selection process**	8	Specify the methods used to decide whether a study met the inclusion criteria of the review, including how many reviewers screened each record and each report retrieved, whether they worked independently, and if applicable, details of automation tools used in the process.	Page 7, Lines 226–241 (Methods: Study Screening and Data Extraction)

Topic	No.	Item	Location where item is reported
**Data collection process**	9	Specify the methods used to collect data from reports, including how many reviewers collected data from each report, whether they worked independently, any processes for obtaining or confirming data from study investigators, and if applicable, details of automation tools used in the process.	Page 7, Lines 226–241 (Methods: Study Screening and Data Extraction)
**Data items**	10a	List and define all outcomes for which data were sought. Specify whether all results that were compatible with each outcome domain in each study were sought (e.g. for all measures, time points, analyses), and if not, the methods used to decide which results to collect.	Page 8, Lines 255–288 (Methods: Statistical Methods)
	10b	List and define all other variables for which data were sought (e.g. participant and intervention characteristics, funding sources). Describe any assumptions made about any missing or unclear information.	Pages 7–8, Lines 226–254 (Methods: Study Screening and Data Extraction)
**Study risk of bias assessment**	11	Specify the methods used to assess risk of bias in the included studies, including details of the tool(s) used, how many reviewers assessed each study and whether they worked independently, and if applicable, details of automation tools used in the process.	Pages 7–8, Lines 242–254 (Methods: Risk of Bias Assessment)
**Effect measures**	12	Specify for each outcome the effect measure(s) (e.g. risk ratio, mean difference) used in the synthesis or presentation of results.	Page 8, Lines 255–288 (Methods: Statistical Methods)
**Synthesis methods**	13a	Describe the processes used to decide which studies were eligible for each synthesis (e.g. tabulating the study intervention characteristics and comparing against the planned groups for each synthesis (item 5)).	Page 8, Lines 255–288 (Methods: Statistical Methods)
	13b	Describe any methods required to prepare the data for presentation or synthesis, such as handling of missing summary statistics, or data conversions.	Page 8, Lines 255–288 (Methods: Statistical Methods)
	13c	Describe any methods used to tabulate or visually display results of individual studies and syntheses.	Page 8, Lines 255–288 (Methods: Statistical Methods)

Topic	No.	Item	Location where item is reported
	13d	Describe any methods used to synthesize results and provide a rationale for the choice(s). If meta-analysis was performed, describe the model(s), method(s) to identify the presence and extent of statistical heterogeneity, and software package(s) used.	Page 8, Lines 255–288 (Methods: Statistical Methods)
	13e	Describe any methods used to explore possible causes of heterogeneity among study results (e.g. subgroup analysis, meta-regression).	Pages 8–11, Lines 255–393 (Methods: Statistical Methods; Results: Subgroup analysis, Lines 370–393)
	13f	Describe any sensitivity analyses conducted to assess robustness of the synthesized results.	Pages 8–12, Lines 255–407 (Methods: Statistical Methods; Results: Sensitivity Analysis, Lines 394–407)
**Reporting bias assessment**	14	Describe any methods used to assess risk of bias due to missing results in a synthesis (arising from reporting biases).	Pages 7–8, Lines 242–254 (Methods: Risk of Bias Assessment); Page 8, Lines 283–286 (Methods: Statistical Methods)
**Certainty assessment**	15	Describe any methods used to assess certainty (or confidence) in the body of evidence for an outcome.	Page 17, Lines 601–605 (Discussion: Strengths and Limitations — formal GRADE certainty assessment not undertaken for observational etiological evidence)
**RESULTS**			
**Study selection**	16a	Describe the results of the search and selection process, from the number of records identified in the search to the number of studies included in the review, ideally using a flow diagram.	Pages 8–9, Lines 290–303 (Results: Overview of the literature screening process)
	16b	Cite studies that might appear to meet the inclusion criteria, but which were excluded, and explain why they were excluded.	Page 9, Lines 295–303 (Results: Overview of the literature screening process)
**Study characteristics**	17	Cite each included study and present its characteristics.	Pages 9–10, Lines 304–319 (Results: Characteristics of Included Studies); Table 3

Topic	No.	Item	Location where item is reported
**Risk of bias in studies**	18	Present assessments of risk of bias for each included study.	Pages 9–10, Lines 304–319 (Results: Characteristics of Included Studies; NOS-xs ratings in Supplementary Table S3)
**Results of individual studies**	19	For all outcomes, present, for each study: (a) summary statistics for each group (where appropriate) and (b) an effect estimate and its precision (e.g. confidence/credible interval), ideally using structured tables or plots.	Table 3 (pages 32–35)
**Results of syntheses**	20a	For each synthesis, briefly summarise the characteristics and risk of bias among contributing studies.	Pages 9–10, Lines 304–319 (Results: Characteristics of Included Studies)
	20b	Present results of all statistical syntheses conducted. If meta-analysis was done, present for each the summary estimate and its precision (e.g. confidence/credible interval) and measures of statistical heterogeneity. If comparing groups, describe the direction of the effect.	Pages 9–12, Lines 320–412 (Results: Meta-analysis results of factors associated with DBM)
	20c	Present results of all investigations of possible causes of heterogeneity among study results.	Page 11, Lines 370–393 (Results: Subgroup analysis)
	20d	Present results of all sensitivity analyses conducted to assess the robustness of the synthesized results.	Pages 11–12, Lines 394–407 (Results: Sensitivity Analysis)
**Reporting biases**	21	Present assessments of risk of bias due to missing results (arising from reporting biases) for each synthesis assessed.	Page 12, Lines 408–412 (Results: Publication bias)
**Certainty of evidence**	22	Present assessments of certainty (or confidence) in the body of evidence for each outcome assessed.	Page 17, Lines 601–605 (Discussion: Strengths and Limitations)
**DISCUSSION**			
**Discussion**	23a	Provide a general interpretation of the results in the context of other evidence.	Page 12, Lines 413–458 (Discussion, 1st paragraph)
	23b	Discuss any limitations of the evidence included in the review.	Pages 16–17, Lines 576–605 (Discussion: Strengths and Limitations)
	23c	Discuss any limitations of the review processes used.	Pages 16–17, Lines 576–605 (Discussion: Strengths and Limitations)

Topic	No.	Item	Location where item is reported
	23d	Discuss implications of the results for practice, policy, and future research.	Pages 15–16, Lines 543–575 (Discussion: Implications for policy and practice)
**OTHER INFORMATION**			
**Registration and protocol**	24a	Provide registration information for the review, including register name and registration number, or state that the review was not registered.	Page 2, Line 54 (Abstract); Page 6, Lines 199–203 (Methods, 1st paragraph)
	24b	Indicate where the review protocol can be accessed, or state that a protocol was not prepared.	Page 6, Lines 199–203 (Methods, 1st paragraph)
	24c	Describe and explain any amendments to information provided at registration or in the protocol.	Page 6, Lines 199–203 (Methods, 1st paragraph)
**Support**	25	Describe sources of financial or non-financial support for the review, and the role of the funders or sponsors in the review.	Page 18, Line 642 (Funding statement)
**Competing interests**	26	Declare any competing interests of review authors.	Page 18, Line 643 (Conflict of interest statement)
**Availability of data, code and other materials**	27	Report which of the following are publicly available and where they can be found: template data collection forms; data extracted from included studies; data used for all analyses; analytic code; any other materials used in the review.	Page 18, Lines 636–640 (Data availability)

From: Page MJ, McKenzie JE, Bossuyt PM, Boutron I, Hoffmann TC, Mulrow CD, et al. The PRISMA 2020 statement: an updated guideline for reporting systematic reviews. MetaArXiv. 2020, September 14. DOI: 10.31222/osf.io/v7gm2. For more information, visit: www.prisma-statement.org
